# Women’s empowerment, intrahousehold influences, and health system design on modern contraceptive use in rural Mali: a multilevel analysis of cross-sectional survey data

**DOI:** 10.1186/s12978-020-01061-z

**Published:** 2021-03-03

**Authors:** Caroline Whidden, Youssouf Keita, Emily Treleaven, Jessica Beckerman, Ari Johnson, Aminata Cissé, Jenny Liu, Kassoum Kayentao

**Affiliations:** 1 Muso, Route de 501 Lodgements SEMA, Bamako, Mali; 2 Department of Disease Control, London School of Hygiene and Tropical Medicine, London, UK; 3 Institute for Social Research, University of Michigan, Ann Arbor, MI USA; 4 Institude for Global Health Sciences, University of California, San Francisco, San Francisco, CA USA; 5 Sub-Direction of Reproductive Health, General Directorate of Health and Public Hygiene, Bamako, Mali; 6 Institute for Health & Aging, University of California, San Francisco, San Francisco, CA USA; 7 Malaria Research & Training Centre, University of Sciences Techniques and Technologies of Bamako, Bamako, Mali

**Keywords:** Contraception, Family planning, Reproductive health, Empowerment, Health systems, Mali, Sub-Saharan Africa

## Abstract

**Background:**

Persistent challenges in meeting reproductive health and family planning goals underscore the value in determining what factors can be leveraged to facilitate modern contraceptive use, especially in poor access settings. In Mali, where only 15% of reproductive-aged women use modern contraception, understanding how women’s realities and health system design influence contraceptive use helps to inform strategies to achieve the nation’s target of 30% by 2023.

**Methods:**

Using household survey data from the baseline round of a cluster-randomized trial, including precise geolocation data from all households and public sector primary health facilities, we used a multilevel model to assess influences at the individual, household, community, and health system levels on women’s modern contraceptive use. In a three-level, mixed-effects logistic regression, we included measures of women’s decision-making and mobility, as well as socio-economic sources of empowerment (education, paid labor), intrahousehold influences in the form of a co-residing user, and structural factors related to the health system, including distance to facility.

**Results:**

Less than 5% of the 14,032 women of reproductive age in our study used a modern method of contraception at the time of the survey. Women who played any role in decision-making, who had any formal education and participated in any paid labor, were more likely to use modern contraception. Women had three times the odds of using modern contraception if they lived in a household with another woman, typically a co-wife, who also used a modern method. Compared to women closest to a primary health center, those who lived between 2 and 5 km were half as likely to use modern contraception, and those between 5 and 10 were a third as likely.

**Conclusions:**

Despite chronically poor service availability across our entire study area, some women—even pairings of women in single households—transcended barriers to use modern contraception. When planning and implementing strategies to expand access to contraception, policymakers and practitioners should consider women’s empowerment, social networks, and health system design. Accessible and effective health systems should reconsider the conventional approach to community-based service delivery, including distance as a barrier only beyond 5 km.

## Résumé

**Contexte:**

Au Mali, où seulement 15% des femmes en âge de procréer utilisent les contraceptifs modernes, la compréhension des réalités des femmes et de la conception du système de santé aident à éclairer les stratégies pour atteindre l’objectif national de 30% d’ici 2023.

**Méthodes:**

En utilisant les données d’enquête de base d’un essai randomisé en grappes, avec la géolocalisation précise de tous les ménages et centres de santé publiques, nous avons utilisé un modèle à plusieurs niveaux pour évaluer l’influence de l’individu, du ménage, de la communauté et du système de santé sur l’utilisation de la contraception moderne. Nous avons utilisé la régression logistique à effets mixtes pour mesurer l’autonomisation et ses sources socio-économiques (éducation, travail rémunéré), les influences intra-ménages sous forme d’une utilisatrice co-résidante et les facteurs structurels liés au système de santé.

**Résultats:**

Moins de 5% des 14 032 femmes en âge de procréer utilisaient la contraception moderne au moment de l’enquête. Les femmes jouant un rôle dans la prise de décision, celles ayant une éducation formelle, un travail rémunéré, étaient plus susceptibles d’utiliser les contraceptifs modernes. Les femmes avaient trois fois plus de chances de faire la contraception moderne si elles vivaient dans un ménage avec une autre femme, généralement une coépouse, qui utilisait une méthode moderne. Comparées aux femmes les plus proches d’un centre de santé, celles qui vivaient entre 2 and 5 kilomètres étaient deux fois moins susceptibles d’utiliser un contraceptif moderne et celles entre 5 and 10 étaient plus susceptibles dans un tiers des cas.

**Conclusions:**

Malgré une faible disponibilité des services dans toute la zone d’étude, certaines femmes—même celles en cohabitation—ont pu surmonter les barrières à l’utilisation des contraceptifs modernes. Lors de la planification et de la mise en œuvre de stratégies pour élargir l’accès à la contraception, les décideurs et les praticiens devraient tenir compte de l’autonomisation des femmes, des réseaux sociaux, et de la conception du système de santé. Les systèmes de santé accessibles et efficaces devraient reconsidérer l’approche conventionnelle de la prestation de services communautaires, en prenant en compte la distance même à moins de 5 kilomètres.

## Plain English Summary

Despite needing methods to avoid unwanted pregnancies and safely space births, many women around the globe are unable to obtain modern contraception (for example, condoms, implants, etc.) particularly in middle and western Africa. In Mali, less than one in six women aged 15 to 49 years use modern contraception. In order to help design strategies to increase use, we need to understand what factors support women to use contraception in settings where access to healthcare is poor. In December 2016 and January 2017, we surveyed 14,032 women in Bankass, rural Mali, and asked them about themselves and their use of reproductive health services, among other topics. Less than five percent used modern contraception at the time of the survey. In a statistical regression analysis, we determined that women who were involved in decisions pertaining to her own health, visiting her relatives, and household spending were more likely to use contraception than those who were not, as were women who had any education and any paid work. Living with another woman in the household who used contraception meant that a woman was three times more likely to use herself. We also found that the further a woman lived from a health center, the less likely she was to use, even within 5 kilometers. When designing and rolling out targeted strategies to expand access to contraception, we ought to consider these elements related to women’s empowerment, intimate relationships, and the broader health system.

## Background

Ensuring access to contraception and women’s family planning needs are met with modern methods is essential to meeting the Sustainable Development Goals related to universal access to reproductive healthcare, gender equality, and the empowerment of women and girls. Among all women of reproductive age globally, the use of modern contraception has increased only marginally between 2000 and 2019 from 42.0 to 44.3%, with the greatest unmet need persisting in middle and western Africa [1]. In Mali, only 15% of women aged 15–49 years used modern contraception at the time of the last Demographic and Health Survey (DHS) in 2018 [2].

Mali’s total fertility rate is among the highest in the world. Women have an average of 6.3 children, with women in rural areas having almost two more children than those in urban areas (6.8 versus 4.9 children per woman) [2]. Although fertility has declined in Mali since 1987 when the average was 7.1 children per woman, certain regions today have fertility rates as high as the 30 year old national average [2]. Despite national policy and law promoting sexual and reproductive health and rights, more than one in five reproductive-aged women in Mali report an unmet need for family planning, including one quarter of women in union and over half of sexually active women not in union [2].

A number of structural barriers may inhibit or delay access to contraception and other basic healthcare services within Mali’s decentralized, pluralistic, fee-for-service healthcare system. Family planning services are theoretically integrated into all levels of public sector care in Mali: national, regional, district, health catchment area, and community. In some communities greater than 5 km from a primary health center (PHC), community health workers (CHW) are stationed in fixed community health posts to provide counseling, services and referral, including for family planning, to patients who seek care and pay the fees for service. However, direct and indirect costs to care, including distance, are well-established barriers to timely, appropriate healthcare across a variety of settings [3–8]. Furthermore, service delivery at all levels of care in Mali suffers from a shortage and inequitable distribution of the health workforce, inadequate clinical mentoring and supervision, and poor infrastructure and frequent stock-outs, which undermine quality of care and patient confidence. Major system-wide reforms in Mali to improve access to care are were announced in February 2019 and expected to take full effect by 2022, including removing user fees for contraceptives and maternal and child health, strengthening the CHW cadre, and increasing national budget allocations to health.

Beyond barriers related to health system design and implementation, women in this context may be further hindered in fulfilling their contraceptive needs due to infringements on their empowerment, defined here as the expansion in people’s ability to make strategic life choices through resources, agency, and achievements [9]. Socio-economic disadvantages such as poor access to formal education and the paid labor force, constraints on physical mobility, limited decision-making power, and gender norms and attitudes have been shown to limit women’s ability to exercise contraceptive choices in settings across sub-Saharan Africa [10–17]. The expansion in women’s ability to make strategic choices related to reproduction in such a prevailing context may be influenced by household composition, familial relationships, and decision-making dynamics. In South Asian settings where extended family ties are strong, intrafamilial influences, such as spousal communications and interactions with mothers-in-law, may play an important role in women’s contraceptive use [18, 19]. In rural Mali, where women in union typically live with their husband’s extended family and 40% are in a polygynous arrangement [2], the role, autonomy, and contraceptive use status of their female household members may expand women’s access to contraceptive choice.

Mali recently developed a renewed five-year national strategic plan for family planning, with the ambitious goal of increasing female modern contraceptive use to 30% by 2023 [20]. Building on the experiences and lessons learned in implementing the previous 5-year plan (2014–2018), the renewed plan for family planning is based on five strategic pillars: demand generation; availability and access to services; supply chain management; an enabling political environment and financing; and monitoring and supervision. In order to achieve this new goal, Mali must attain a rapid growth rate in contraceptive use comparable only to that achieved by Sierra Leone in the West African region [20]. Further elucidating how health system design and women’s realities influence modern contraceptive use helps to determine how such ambitious plans should be operationalized in order to improve access.

In this study, we aim to: (1) describe modern contraceptive use among women of reproductive age in the under-studied, high fertility, rural Malian context of the Bankass district, including methods and procurement among users; (2) explore descriptively and visually household and village composition of reproductive-aged women and their use of modern contraception; and (3) identify predictors of modern contraceptive use in this context where utilization is exceptionally low. We use a multilevel modeling approach using detailed household survey data, including geolocated measures of distance, to assess influences on women’s modern contraceptive use at the individual, household, community, and health system levels. We include ‘direct’ measures of women’s empowerment (decision-making and mobility in the public domain), as well as indirect socio-economic sources of empowerment (education and paid labor force participation). We explore the role of intimate female social networks by assessing how living in a household with another woman who uses modern contraception influences adoption. Determining what structural barriers to dismantle and social relationships to leverage in order to expand contraceptive access is key to meeting national and international goals for women’s wellness, health, and survival.

## Methods

### Study design

We conducted a cross-sectional household survey in the communities of seven health catchment areas of the rural Bankass district, Mali from December 2016 to January 2017. This survey served as the baseline for an ongoing cluster-randomized controlled trial (trial registration number NCT02694055; N = 137 village-clusters) to assess the effects of a proactive approach to community-based healthcare delivery on child mortality and access to care over three years, including access to modern and long-acting reversible contraception (secondary trial endpoint) [21]. Here, we analyze baseline survey data from women of reproductive age to assess contraceptive use before the launch of intervention activities.

### Study setting

The Bankass health district is part of the Mopti region in central Mali, approximately 600 km northeast of the nation’s capital, Bamako. The district has a population of approximately 300,000 people and is served by a public, secondary referral hospital located in Bankass, the largest town in the district [22]. It was chosen for the trial in collaboration with the Malian Ministry of Health and Social Affairs based on high under-five mortality and low healthcare utilization in the region, similar to other rural Malian settings [23], as well as few concurrent health interventions in the district and interest from local authorities to collaborate. Within the Bankass health district, the study was conducted in seven (of 22) contiguous, rural health catchment areas: Dimbal, Doundé, Ende, Kani Bozon, Koulongon, Lessagou, and Soubala, an area with a population of approximately 100,000 people. Each health catchment area is served by a public sector PHC.

### Study participants

In the context of rural Mali, extended families often live together in family compounds comprised of multiple households. Our survey definition of a household within a family compound was a monogamous or polygynous marital arrangement with or without children and additional relatives, or a single mother with or without additional relatives. All women aged 15 to 49 years permanently residing in the study area with no plans to leave during the trial period and who provided consent or assent were eligible to participate in the women’s questionnaire component of the household survey. From the present analysis we excluded all women who reported being pregnant at the time of the survey (N = 2022) or who reported having reached menopause or having had a hysterectomy (N = 299).

### Data sources and measurement

We adapted our household survey instrument (see Additional File 1) from the Mali DHS and programmed it in Open Data Kit. The survey captured detailed information on household and individual socio-demographic characteristics, utilization of reproductive, maternal and child health services, and recorded household geographic coordinates, among other topics. All surveyors were women who were not members of the villages they surveyed, due to the sensitive nature of questions related to contraception and reproductive health. Respondents participated in French, Bamanankan, Peulh, or the Dogon dialects of Tomokan and Tingu.

### Measures

#### Outcomes

We evaluated women’s self-reported use of a modern method of contraception at the time of the survey. We defined modern methods according to the World Health Organization (WHO) [24] and Malian guidelines, and included female and male sterilization, female and male condoms, intrauterine device (IUD), implant, injectable contraceptive, oral contraceptive pill (OCP), diaphragm, foam/spermicidal jelly, the lactational amenorrhea method (LAM), and the standard days method (e.g., cycle beads). Traditional methods included the rhythm/calendar method, withdrawal, herbal, and other methods. For women who reported using multiple methods concurrently (N = 5), the more efficacious method was chosen for analysis (i.e., sterilization > implant > IUD > injectable > other modern method > traditional method).

We descriptively examined length of use and place and cost of last procurement among all contraceptive users. Length of contraceptive use in months was calculated by subtracting the month and year that the woman reported using the current method without interruption from the month and year of the survey. Initiation month was assigned between one and 12 at random using the *runiform* function in Stata 15 if it was missing (N = 123/710). The place where the current method was last procured was categorized as within the health sector or outside the health sector. Within the health sector included national, regional, or district hospitals, PHCs, CHWs, and private clinics. Outside the health sector included at home, at boutiques, kiosks, bars, or nightclubs, black market vendors, or personal contacts.

#### Predictors

We elaborated a list of potential predictors a priori based on existing evidence and contextual knowledge. At the individual level, these included: women’s age (5-year categories); number of living children (none, one or two, three or four, five or six, seven or more); marital status (monogamous, polygynous, not currently married); tolerant attitudes for spousal violence (coded any tolerance versus none, based on whether she believed a husband was justified in hitting or beating a wife under any of the seven circumstances evaluated, including if she used contraception without his consent); education (any formal schooling versus none); participation in paid labor (any versus none); and empowerment measures we adapted from existing scales, including mobility and decision-making power. We coded women’s mobility categorically based on their having ever been to the market place, health center, women’s group, or outside the village (never been to any, been to some or all but never alone, been to at least one alone—with which we capture independent mobility) [25]. Women were coded as having any involvement in decision-making versus none, based on whether they reported making decisions, either independently or jointly with someone else in the family, for any of the three domains asked (i.e., her own healthcare, visiting her relatives, household purchasing) [26].

Household level predictors included: another woman of reproductive age in the household using modern contraception; household wealth quintiles constructed using principal components analysis of asset indicators; [27] household food insecurity (coded as any versus none, based on whether the respondent affirmed that in the last 30 days, there was no food to eat due to a lack of resources, or someone went to sleep hungry because there was not enough food, or someone went a whole day and night without eating because there was not enough food); [28] and household distance to nearest public sector health facility. Orthodromic (great-circle) distance estimates were based on Geographic Information System (GIS) data for the entrance to the family compound, each PHC, and the district referral hospital. When GIS data for the family compound was missing (N = 560), we approximated household distance using GIS collected at the central gathering place in the village. We included a community level factor for the availability of CHW services at the time of the survey (coded as having a CHW posted in the village or hamlet at a fixed community health site, having a CHW provide services in the village or hamlet but not posted there, or not having any CHW services available), based on documentation from the Ministry of Health and Social Affairs.

### Statistical analysis

All statistical analyses were performed using Stata version 15 (Stata Corporation, Texas, USA).

#### Descriptive data

We first examined descriptively sample characteristics and contraceptive use outcomes. Categorical sample characteristics were calculated as proportions of all women in the sample (i.e., including those with missing characteristics data). The main outcome was calculated among those with non-missing modern contraceptive use data. We compared sample characteristics between those with and without missing data on the main outcome. For continuous variables, we calculated summary statistics appropriate to the variable distribution in the sample population (e.g., mean, median).

We georeferenced the concession location data using OpenStreetMap. Kernel Density Estimation was employed to generate a density raster (heatmap) in QGIS v3.4.7 and to visualize the spatial/geographic clustering of women using modern contraception as well as multi-user households and village-clusters with no users, across the study area. The radius was set at 0.01 map unit.

Within households that had multiple women of reproductive age where some but not all were using modern methods of contraception, we explored descriptively how, within the same household, users compared with non-users in terms of their socio-demographic characteristics, role in the household, and empowerment measures. We also explored descriptively how household level factors, including household decision-making dynamics, compared between households where there was at least one modern contraceptive user and households where there were none.

#### Regression analysis

We conducted a multilevel regression analysis to assess factors at multiple levels influencing modern contraceptive use among women of reproductive age. As the percent missing on outcome data and covariates was small, these observations were dropped from the regression analysis. Due to the clustering of female modern contraceptive users within households, family compounds, village-clusters, and health catchment areas, we employed a multilevel modeling approach. We used a three-level, mixed-effects logistic regression with random effects at the family compound and village-cluster levels, and fixed effects for health catchment area in order to adjust for any time-invariant unobserved heterogeneity across catchment areas, such as availability of contraceptive methods or characteristics of provision at the PHCs.

$${\text{Level 1}}: \eta_{ijk} = \alpha_{0jk} + \beta_{1} X_{ijk} + \varepsilon_{ijk}$$

$${\text{Level 2}}:\alpha_{0jk} = \delta_{00k} + \gamma_{1} Z_{0jk} + \mu_{0jk}$$

$${\text{Level 3}}: \,\delta_{00k} = \theta_{000} + \lambda_{1} C_{00k} + \upsilon_{00k}$$

The Level 1 equation represents variation at the individual woman level. ${\eta }_{ijk}=$ $log\left(\frac{{\pi }_{ijk}}{1-{\pi }_{ijk}}\right)$, ${\pi }_{\mathrm{ijk}}$ denotes the probability that the *ith* woman in the *jth* family compound and the *kth* village-cluster uses modern contraception. ${X}_{ijk}$ denotes a vector of individual woman-level and household-level (e.g., wealth, food insecurity) variables of interest, and ${\beta }_{1}$ represents the coefficients for this set of covariates. ${\varepsilon }_{ijk}$ is the woman-level error term, with variance ${\sigma }_{(1)}^{2}$. The Level 2 equation represents variation at the level of the family compound, where ${\alpha }_{0jk}$ is a function of: ${Z}_{0jk}$, which denotes family compound-level covariates (i.e., distance to the nearest public sector health facility); ${\delta }_{00k}$, which is a systematic component modelled as the compound specific random intercept; and ${\mu }_{0jk}$, representing the family compound-level random effect with variance ${\sigma }_{(2)}^{2}$. The Level 3 equation represents variation at the level of the village-cluster. ${C}_{00k}$ denotes cluster-level covariates (i.e., availability of CHW services), ${\theta }_{000}$ represents the village-cluster specific intercept, and ${\upsilon }_{00k}$ is the village-cluster level random effect with variance ${\sigma }_{(3)}^{2}$.

We estimated adjusted odds ratios (AORs) and reported 95% confidence intervals (CIs). For all categorical variables, we conducted a likelihood ratio test to assess the evidence of an association between the variable and the outcome.

#### Sensitivity analyses

To assess the extent to which missing outcome data affected the results, we ran the model assuming women with missing contraceptive use data were all not modern contraceptive users, and again assuming they were all modern contraceptive users.

## Results

### Sample characteristics

Our sample included 10,872 households within 4987 family compounds, with a median household size of six members (IQR 4, 9) (Table 1). Median household distance to the nearest health center was 5.41 km. These households included 14,032 women of reproductive age (Table 2), with a median age of 30 years. Nine out of ten women (90.4%) were married; 38.6% were in a polygynous marital arrangement and 51.8% in a monogamous arrangement. Women had a median of three living children (IQR 1, 5). Only one in ten women (10.7%) had received any formal education and only slightly more (13.7%) participated in any paid labor. Approximately one quarter (26.9%) participated to any extent in decision-making; this was the same for women in monogamous (26.3%) and polygynous (26.7%) marital arrangements.

**Table 1:** Household level sample characteristics for women of reproductive age by modern contraceptive use status

Household level characteristic	Modern users N = 626		Non modern users N = 13,357		All women N = 14,032	
	n	%	n	%	n	%
Household size						
Median/IQR	6	4, 9	6	4, 9	6	4, 9
Missing	1	0.2	27	0.2	28	0.2
Distance to health center						
< 2 km	243	38.8	2455	18.4	2708	19.3
2–4.99 km	149	23.8	3327	24.9	3489	24.9
5–6.99 km	104	16.6	3159	23.7	3273	23.3
7–9.99 km	52	8.3	2527	18.9	2584	18.4
≥ 10	61	9.7	1426	10.7	1494	10.7
Missing	17	2.7	463	3.5	484	3.5
Household wealth quintile^a^						
Poorest	85	13.6	2294	17.2	2382	17.0
Poor	108	17.3	2479	18.6	2591	18.5
Middle	111	17.7	2589	19.4	2709	19.3
Rich	139	22.2	2844	21.3	2996	21.4
Richest	175	28.0	2983	22.3	3178	22.7
Missing	8	1.3	168	1.3	176	1.3
Water and sanitation^b^						
Unimproved toilet facilities	235	37.5	6869	51.4	7127	50.8
Improved toilet facilities	388	62.0	6437	48.2	6851	48.8
Missing	3	0.5	51	0.4	54	0.4
Unimproved water source	150	24.0	6118	45.8	6284	44.8
Improved water source	476	76.0	7237	54.2	7746	55.2
Missing	0	0	2	0.0	2	0.0
Food insecurity in past 30 days						
Little to no hunger in the household	554	88.5	11,685	87.5	12,280	87.5
Moderate hunger in the household	45	7.2	932	7.0	980	7.0
Severe hunger in the household	25	4.0	725	5.4	755	5.4
Missing	2	0.3	15	0.1	17	0.1
CHW services available						
None	469	74.9	9148	68.5	9649	68.8
Satellite village^c^	33	5.3	1868	14.0	1908	13.6
Posted village	124	19.8	2341	17.5	2475	17.6
Health catchment area						
Dimbal	89	14.2	3051	22.8	3145	22.4
Lessagou	111	17.7	2083	15.6	2195	15.6
Doundé	68	10.9	1684	12.6	1756	12.5
Ende	89	14.2	639	4.8	728	5.2
Soubala	35	5.6	2371	17.8	2407	17.2
Kanibozon	128	20.5	1302	9.8	1448	10.3
Koulongon	106	16.9	2227	16.7	2353	16.8

^a^Household wealth quintile here excludes water, sanitation, and hygiene measures, which are reported separately

^b^We used the WHO/UNICEF Joint Monitoring Programme for Water Supply and Sanitation harmonized survey questions and definitions to define improved drinking water and improved sanitation [39]

^c^Satellite villages are within the CHW’s catchment area, 5 km or less of the village where the CHW has a fixed site (posted village)

**Table 2:** Individual level sample characteristics for women of reproductive age by modern contraceptive use status

Individual level characteristic	Modern users N = 626		Non modern users N = 13,357		All women N = 14,032	
	n	%	n	%	n	%
Age						
15–19	49	7.8	1770	13.3	1839	13.1
20–24	138	22.0	2232	16.7	2377	16.9
25–29	120	19.2	2620	19.6	2746	19.6
30–34	135	21.6	2308	17.3	2447	17.4
35–39	99	15.8	1894	14.2	2998	14.2
40–44	58	9.3	1440	10.8	1499	10.7
45–49	27	4.3	1093	8.2	1126	8.0
Number of living children						
Median/IQR	3	1, 5	3	1, 5	3	1, 5
None	76	12.1	2284	17.1	2376	16.9
1–2	184	29.4	3656	27.4	3858	27.5
3–4	157	25.1	3513	26.3	3674	26.2
5–6	128	20.5	2555	19.1	2688	19.2
7 +	79	12.6	1331	10.0	1416	10.1
Missing	2	0.3	18	0.1	20	0.1
Ethnicity						
Dogon	585	93.5	12,024	90.0	12,646	90.1
Peulh	14	2.2	1012	7.6	1036	7.4
Other	27	4.3	320	2.4	350	2.5
Religion						
Muslim	597	95.4	13,087	98.0	13,732	97.9
Catholic	19	3.0	162	1.2	181	1.3
Other	10	1.6	108	0.8	119	0.9
Marital status						
Never married	36	5.8	1131	8.5	1179	8.4
Divorced/widowed	4	0.6	158	1.2	162	1.2
Polygynous marriage	246	39.3	5141	38.5	5409	38.6
Monogamous marriage	340	54.3	6917	51.8	7272	51.8
Married, arrangement unspecified	0	0	10	0.1	10	0.1
Education						
None	457	73.0	12,016	90.0	12,515	89.2
Primary	120	19.2	1191	8.9	1318	9.4
Secondary or higher	47	7.5	134	1.0	181	1.3
Missing	2	0.3	16	0.1	18	0.1
Participates in paid labor						
No	458	73.2	11,608	86.9	12,111	86.3
Yes	168	26.8	1746	13.1	1917	13.7
Missing	0	0	3	0.0	4	0.0
Mobility						
Been to no place	241	38.5	4517	33.8	4767	34.0
Been to some/all places but none alone	90	14.4	2404	18.0	2498	17.8
Been to some places alone	196	31.3	3870	29.0	4094	29.2
Been to all places alone	97	15.5	2532	19.0	2634	18.8
Missing	2	0.3	34	0.3	39	0.3
Tolerant attitudes for spousal violence						
Never tolerated	168	26.8	3677	27.5	3853	27.5
Sometimes tolerated	280	44.7	5680	42.5	5991	42.7
Always tolerated	163	26.0	3519	26.4	3687	26.3
Missing	15	2.4	481	3.6	501	3.6
Decision-making						
Not involved in any domains	421	67.3	9747	73.0	10,210	72.8
Involved in some domains	142	22.7	2318	17.4	2465	17.6
Involved/independent in all domains	61	9.7	1242	9.3	1204	9.3
Missing	2	0.3	50	0.4	53	0.4

Just 49 women (0.4%) were missing main outcome data on contraceptive use at the time of the survey and were therefore excluded from subsequent analyses. A relatively larger proportion of these women were Peulh, young (15 to 19 years), never married, nulliparous, having independent mobility, not involved in decision-making and in wealthier households compared to those with non-missing contraceptive use data (See Supplementary Tables 1 and 2, Additional File 2).

### Descriptive results

**Fig. 1:**
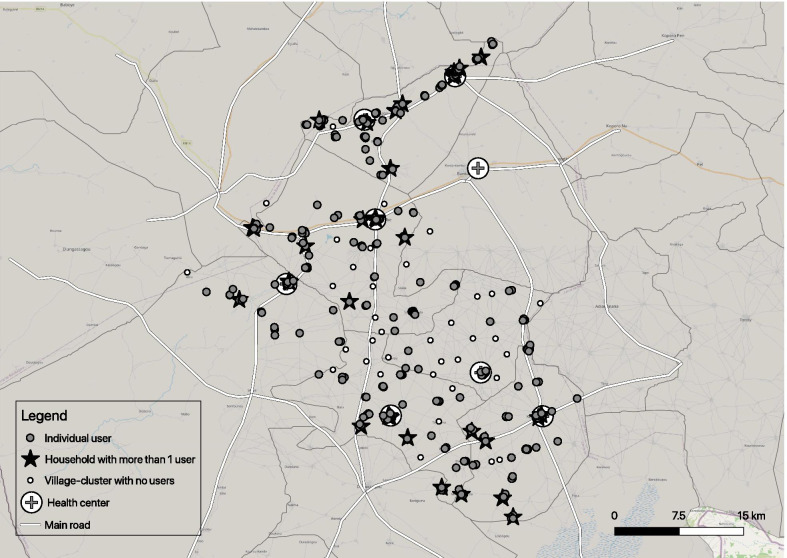
Map of modern contraceptive users across the study area, using precise geographic coordinates collected during the survey

The geographic distribution of female modern contraceptive users across the study area is depicted in Fig. 1. Approximately a third of village-clusters (N = 44/137) had no modern contraceptive users at all (blank circles, Fig. 1) and 51.8% (N = 71/137) had more than one user. More than a quarter of all women who lived with a modern contraceptive user in the same household also used modern contraception (N = 85/324). Put another way, 14% of all female modern contraceptive users lived with another modern contraceptive user in the same household (N = 85/626).

Table 3 represents household composition of reproductive-aged women and their use of modern contraception. Among households that had more than one woman of reproductive age (N = 2640/10,872; column 4, Table 3), less than 2% (N = 42/2640) had more than one woman who used modern contraception (stars, Fig. 1). One household had three users (a mother and her two daughters) and the rest had two users, most commonly first and second co-wives (N = 30/41), followed by other pairings of co-wives in households with three or four wives (7/41) and mother-daughter dyads (4/41). Within multi-woman households in which some but not all women were using modern contraception (N = 195 households containing 446 women; Table 3), those who were not using tended to be younger, unmarried and nulliparous (results not shown). Households with at least one female modern contraceptive user (N = 584 households containing 867 women; Table 3) tended to be richer, closer to the nearest health center, and more inclusive of women in decision-making than households with none (results not shown).

**Table 3:** Household composition of women of reproductive age and their use of modern contraception

	Among WRA N = 14,032 n (%)	Among HHs N = 10,872 n (%)	Among multi-WRA HHs N = 2640 n (%)
Household with no users	**13,140 (93.6)**	**10,265 (94.4)**	**2412 (91.4)**
No user in a single-woman household	7853 (56.0)	7853 (72.2)	NA
No users in a multi-woman household	5287 (37.7)	2412 (22.2)	2412 (91.4)
Household with one user	**765 (5.5)**	**542 (5.0)**	**184 (7.0)**
User in a single-woman household	358 (2.6)	358 (3.3)	NA
User in a multi-woman household	407 (2.9)	184 (1.7)	184 (7.0)
Household with more than one user	**102 (0.7)**	**42 (0.4)**	**42 (1.6)**
Some users in a multi-woman household^a^	39 (0.3)	11 (0.1)	11 (0.4)
All users in a multi-woman household^b^	63 (0.5)	31 (0.3)	31 (1.2)
Missing	**25 (0.2)**	**23 (0.2)**	

^a^Precisely, 2 women using modern contraception within a household of 3, 4 or 5 reproductive-aged women;

^b^Precisely, 2 women using modern contraception within a household of 2 reproductive-aged women (N = 30 households) or 3 women using modern contraception within a household of 3 reproductive-aged women (N = 1 household);

*HH* household; *WRA* women of reproductive age

Less than 5% of women (4.5%) reported using any method of contraception at the time of the survey, ranging from 1.5 to 12.2% across health catchment areas, the vast majority (98.6%) of whom used a modern method (Table 4). Among women who used modern methods, half used the injectable contraceptive (49.7%), one quarter used the implant (26.2%), 15.3% the OCP, and 5.1% the IUD. Five women used two options concurrently: four used the injectable with another method (two with implant, one with OCP, one with rhythm/calendar method) and one used the OCP with jelly. Over three quarters of all contraceptive users (78.4%) most recently acquired their method at the PHC. The method most commonly procured outside the health sector was the OCP (19.8% of all OCP users; 52.6% of whom procured from black market vendors). The median cost of the most recent procurement was 0.81 USD (IQR 0.32, 1.61). The least expensive method was the OCP (0.16 USD) and among the most expensive was the implant (1.21 USD).

**Table 4:** Methods and characteristics of contraceptive use among women of reproductive age

Method	Current utilization			Last procurement for current method			
	n	%	Median length^a^ of use in months (IQR)	Median cost^a,d^ in USD (IQR)	Site^a^ %		
					PHC	Other^e^ health sector	Outside^f^ health sector
Any method	635	4.5^b^	14 (8, 34)	0.81 (0.32, 1.61)	78.4	13.6	8.1
Traditional method	9	1.4	21 (13, 22)	1.21 (0.40, 2.54)	87.5	0	12.5
Modern^c^ method	626	98.6	14 (8, 34)	0.81 (0.32, 1.61)	78.2	13.8	8.0
Injectable	311	49.7	14 (7.5, 34)	0.81 (0.81, 1.61)	82.0	10.9	7.1
Implant	164	26.2	13 (7, 33)	1.21 (0.00, 4.03)	78.7	17.7	3.7
OCP	96	15.3	17 (8, 47)	0.16 (0.16, 0.81)	64.6	15.6	19.8
IUD	32	5.1	16.5 (11.5, 37)	0.81 (0.16, 3.23)	80.7	16.1	3.2
Other modern	23	3.7	17.5 (9, 25)	1.21 (0.00 1.61)	78.3	13.0	8.7

^a^Summary statistics are among those with complete/non-missing length or cost or site data. Missing year of initiation data: N = 13 total users; N = 7 OCP users; N = 3 injectable users; N = 2 implant users; N = 1 other modern method users. Missing cost of method: N = 17 total users; N = 6 implant users; N = 4 IUD users; N = 3 injectable users; N = 2 other modern method users; N = 1 OCP users; N = 1 traditional method users. Missing location of last procurement: N = 2 total users; N = 1 IUD users; N = 1 traditional method users

^b^Percentage among the 13,983 women (99.6% of the 14,032 in the sample population) who had complete/non-missing outcome data on contraceptive use. Percentage ranged by health catchment area: 1.5% in Soubala; 2.8% in Dimbal; 3.9% in Doundé; 4.5% in Koulongon; 5.2% in Lessagou; 9.4% in Kanibozon; 12.2% in Ende

^c^Modern is defined according to WHO definition, which includes male and female sterilisation, intrauterine device (IUD), implant, injectable, oral contraceptive pill (OCP), male and female condom, diaphragm, jelly, cycle beads, and lactational amenorrhea method (LAM)

^d^Cost converted at the approximate rate at the time of the survey: 620FCFA per 1USD

^e^The majority procured from the district referral hospital in Bankass (41.9%; 36/86) or CHW (34.9%; 30/86) or private clinic (11.6%; 10/86)

^f^The majority procured from home (43.1%; 22/51) or black market vendors (25.5%; 13/51) or boutiques (13.7%; 7/51)

### Regression results

Ninety-five percent of observations (N = 13,376 complete cases) were retained in the regression analysis (Table 5). Women had more than three times the odds of using modern contraception if they had any formal education (AOR 3.28; 95% CI 2.52, 4.27), and were 71% more likely to use modern contraception if they participated in any paid labor (AOR 1.71; 95% CI 1.35, 2.17). Living in the same household as another woman who used modern contraception was the strongest factor influencing modern contraceptive use after education (AOR 3.04; 95% CI 1.95, 4.73). Women were 29% more likely to use modern contraception if they exerted any, even shared, power over decision-making (AOR 1.29; 95% CI 1.04, 1.60); this was after controlling for all covariates, including women’s education and paid labor force participation. Women who reported some mobility but none independently were 42% less likely to use modern contraception compared to women who reported no mobility at all (AOR 0.58; 95% CI 0.42, 0.79). There was no evidence of an association between women’s independent mobility or tolerant attitudes for spousal violence and modern contraceptive use.

**Table 5:** Three-level mixed-effects logistic regression modeling associations between individual, household, and community level factors and women’s modern contraceptive use

Variables	n	%	Adjusted OR (95% CI)	p value^b^
Individual level				
Age in years				< 0.0001
15–19	1839	13.1	1.0	Ref
20–24	2377	16.9	2.37 (1.52, 3.68)	< 0.001
25–29	2746	19.6	1.89 (1.18, 3.04)	0.008
30–34	2447	17.4	2.29 (1.39, 3.77)	0.001
35–39	2998	14.2	1.97 (1.17, 3.32)	0.010
40–44	1499	10.7	1.31 (0.75, 2.27)	0.342
45–49	1126	8.0	0.80 (0.43, 1.49)	0.485
Number of living children				0.0053
None	2376	17.0	1.0	Ref
1–2	3858	27.5	1.52 (1.05, 2.20)	0.028
3–4	3674	26.2	1.42 (0.95, 2.13)	0.084
5–6	2688	19.2	1.60 (1.04, 2.46)	0.031
7 +	1416	10.1	2.39 (1.49, 3.83)	< 0.001
Marital status				0.0501
Not currently married	1341	9.6	1.0	Ref
Polygynous	5409	38.6	1.61 (0.97, 2.67)	0.067
Monogamous	7272	52.9	1.79 (1.09, 2.92)	0.020
Education				
None	12,515	89.3	1.0	Ref
Any	1499	10.7	3.28 (2.52, 4.27)	< 0.001
Participates in paid labor				
No	12,111	86.3	1.0	Ref
Yes	1917	13.7	1.71 (1.35, 2.17)	< 0.001
Mobility				0.0028
Been to no place	4767	34.1	1.0	Ref
Been to any place but none alone	2498	17.9	0.58 (0.42, 0.79	0.001
Been to any place alone	6728	48.1	0.82 (0.64, 1.03)	0.093
Spousal violence				
Not tolerated	3853	28.1	1.0	Ref
Tolerated	9872	71.9	1.00 (0.80, 1.25)	0.978
Decision-making				
None	10,210	73.0	1.0	Ref
Any	3769	27.0	1.29 (1.04, 1.60)	0.019
Household level				
Someone else in the household using modern contraception				
No	13,682	97.7	1.0	Ref
Yes	324	2.3	3.04 (1.95, 4.73)	< 0.001
Distance to health center^c^				< 0.0001
< 2 km	2859	20.4	1.0	Ref
2–4.99 km	3586	25.6	0.50 (0.33, 0.75)	0.001
5–6.99 km	3329	23.7	0.33 (0.20, 0.53)	< 0.001
7–9.99 km	2677	19.1	0.33 (0.19, 0.55)	< 0.001
≥ 10	1581	11.3	0.70 (0.38, 1.31)	0.266
Food insecurity				
None	11,931	85.1	1.0	Ref
Any	2084	14.9	1.05 (0.78, 1.41)	0.745
Wealth quintile^d^				0.4582
Richest	2385	17.2	1.0	Ref
Rich	2592	18.7	1.04 (0.79, 1.37)	0.788
Middle	2689	19.4	0.91 (0.68, 1.23)	0.554
Poor	2997	21.6	0.86 (0.64, 1.18)	0.354
Poorest	3193	23.0	0.78 (0.55, 1.09)	0.140
Community level				
CHW services available				0.2920
None	9649	68.8	1.0	
Satellite village	1908	13.6	0.81 (0.48, 1.39)	0.452
Posted village	2475	17.6	1.27 (0.82, 1.97)	0.291
Health catchment area^e^				< 0.0001
Dimbal	3145	22.4	1.0	Ref
Lessagou	2195	15.6	1.90 (1.14, 3.17)	0.014
Doundé	1756	12.5	1.75 (1.00, 3.07)	0.051
Ende	728	5.2	3.28 (1.67, 6.44)	0.001
Soubala	2407	17.2	0.56 (0.30, 1.03)	0.063
Kanibozon	1448	10.3	4.05 (2.36, 6.97)	< 0.001
Koulongon	2353	16.8	1.86 (1.10, 3.14)	0.020
Random effects				
Village-cluster level (level three) variance (SD)			0.22 (0.09)	
Compound-within-cluster level (level two) variance (SD)			0.98 (0.29)	
Level three ICC (95% CI)			0.05 (0.02, 0.10)	
Level two ICC (95% CI)			0.27 (0.18, 0.38)	
Log likelihood			− 2081.39	

^a^N = 13,376 complete cases, or 95% of all women in the analytic sample

^b^Value provided in line with the categorical variable name is the result of the likelihood ratio test

^c^Village distance to health center substituted if household distance to health center was missing (N = 560)

^d^Household wealth quintile in the regression models includes water, sanitation, and hygiene measures, as these are not explored separately

^e^Largest health catchment area in terms of sample population is used as the reference category

There was very strong evidence (p < 0.0001) that greater distance to a public sector health facility reduced the odds of modern contraceptive use. Compared to women who lived less than 2 km from a health center, those who lived between 2 and 5 km were 50% less likely to use modern contraception (AOR 0.50; 95% CI 0.33, 0.75); those who lived between 5 and 10 km were 67% less likely (AOR 0.33; 95% CI 0.19, 0.55); and those who lived 10 km or more were 30% less likely (AOR 0.70; 95% CI 0.38, 1.31). The strength of the evidence declined in the group farthest away, where approximately 70% were serviced by a CHW compared to 45% for those between 5 and 10 km. Controlling for distance to health center and all other covariates, there was no evidence (p = 0.2920) that having CHW services available in the community as a base or satellite site was associated with modern contraceptive use. The intracluster correlation coefficient (ICC) at the village-cluster level was 0.05 (95% CI 0.02, 0.10) and the family compound-within-cluster ICC was 0.27 (95% CI 0.18, 0.38).

The odds of using a modern method of contraception were greater if the woman was in a polygynous (AOR 1.61; 95% CI 0.97, 2.67) or monogamous (AOR 1.79; 95% CI 1.09, 2.92) marital arrangement than if she was not currently married. Both age (p < 0.0001) and number of living children (p = 0.0053) were also significant predictors in the model. Regression results were consistent in sensitivity analyses (results not shown).

## Discussion

Our study in seven health catchment areas of the Bankass district in the Mopti region of Mali found a modern contraceptive prevalence below 5%. This is similar to, but even lower than the 8.7% regional average in 2018 (an increase from 2.7% in 2012–2013 [23]), despite over a third of all women in Mopti desiring family planning [2]. Another study in the Youwarou district of Mopti found 8.8% of non-pregnant, reproductive-aged women visiting PHCs used modern contraception [29]. The injectable contraceptive was the most common method used in our study population, followed by the implant, which were also the two most common in the 2018 DHS (34% and 44%, respectively) [2]. Anecdotally, there is a preference for these methods in our context due to their long-acting and discrete nature. We may have had underreporting of traditional methods, although the DHS also reports that less than 1% of all contraceptive users used traditional methods [2].

Such low modern contraceptive prevalence may be partly explained by the services available. Within a global context of shortages and inequitable distribution of human resources for health, approximately 37% of doctors, nurses, and midwives in Mali work in rural areas where three quarters of the population resides [20]. Where healthcare workers are available in Mali’s rural areas, distance, quality, and cost create barriers to basic health services [8]; contraceptive options can be limited and stock-outs frequent. Yet, despite chronically poor service availability and accessibility across our entire study area, some women—and even some pairings of women within single households—used modern contraception. Distilling individual, household, community, and health system level factors associated with contraceptive use in this context helps to inform the design of strategies to reduce unmet need for contraception where access is at its absolute worst.

We found that women who played any role in decision-making, who had any formal education, and participated in any paid labor, were more likely to use modern contraception. In addition, a greater percentage of households that had at least one modern contraceptive user included any reproductive-aged woman in decision-making, compared to households that had no users (37% versus 29%). We found unexpected results related to women’s mobility, where women with some mobility were less likely to use modern contraception than those who had none. Our findings on the association between women’s education and contraceptive use are consistent with other studies from sub-Saharan Africa [10–12, 17]. The evidence base for the role of women’s empowerment, as measured by decision-making and mobility, on contraceptive use is mixed and dominated by research conducted in South Asia [17, 18, 26, 30]. Our results suggest that having any involvement in decision-making related to healthcare, visiting relatives, or household purchasing more adequately captured women’s capabilities to make strategic choices related to contraceptive use in this context than having freedom of movement to the marketplace, health center, women’s group, or outside the village. It may be that having ever been or been alone to these places does not reflect a woman’s physical autonomy in this context, but rather their availability or distribution. Alternatively, it may be that having recently (rather than ever) been to these places—as mobility is so dependent on age or phase of life [30]—would be a more appropriate predictor of current contraceptive use.

Living in the same household as another woman who used modern contraception was strongly associated with an individual’s uptake in our study. Our findings contribute to the broader healthcare utilization literature on the importance of engaging social networks including in Mali [31, 32], by illustrating the power of intimate intrahousehold female relations—among cowives, and among mothers and their daughters—in influencing contraceptive use. One woman’s ‘functioning achievement’ [9] in accessing modern contraception that she desires may transform the intrahousehold context in which another woman makes a strategic life choice to use. These findings, taken together with education, paid labor, and decision-making, suggest that utilizing contraceptive services in this poor access, low use context may have required considerable assertiveness on the part of women. Strategies to expand women’s ability to make contraceptive choices might engage direct axes of empowerment, sources of empowerment, and the settings for empowerment [33]—decision-making, education and paid labor, and intrahousehold female relations, in this context.

Health systems must be designed to meet women most of the way. Distance to nearest public sector health facility, where 85% of contraceptive users procured their method, was a strong predictor of modern contraceptive use. Compared to women closest to a health center, those who lived between 2 and 5 km were half as likely to use modern contraception, and those between 5 and 10 km were a third as likely. A growing body of literature suggests that even relatively short distances from health facilities are associated with adverse health outcomes [34]; however, the 5 km cut-off continues to dominate research, policy, and practice [7]. Although CHWs offered family planning counseling and services in some villages 5 km or more from a PHC at the time of the survey, only condoms and the OCP were offered and women were referred to the more distant health centers for other methods. CHW capabilities to deliver a range of specific health interventions and contribute to health outcomes, including contraceptive use, is well established [35]—when CHW programmes are appropriately designed and implemented, and supported by health system enablers [36]. In our study setting, CHWs services were accessible only to patients who initiated their own care-seeking from the fixed community health post, and who paid a fee for service—a practice known to hinder utilization across settings and interventions. Our findings suggest that this conventional approach to CHW service delivery is insufficient to increase contraceptive use. Home visits by CHWs have shown particular promise as an alternative approach to community-based contraceptive service delivery [37, 38]

Finally, variation in modern contraceptive prevalence and methods between PHC catchment areas and the parameter estimates for PHC catchment areas in the regression model suggest that the availability and quality of contraceptive services differed in important ways between the seven neighboring PHCs in the Bankass district. The intracluster correlation coefficients (ICC) in the multilevel model indicated that the local village-cluster environment and the family compound environment within a given village-cluster played a role in contraceptive use in addition to the individual, household, and health catchment area fixed effects. We note that the two catchment areas with the highest prevalence of modern contraceptive use were the smallest in terms of population and tended to be wealthier, and anecdotally, are better connected to societal resources through tourism.

Our study was subject to some important limitations. First, we were unable to measure unmet need for contraception and thus analyzed use among all women of reproductive age. We were unable to exclude women intending to become pregnant at the time of the survey, as respondents did not report this data. Furthermore, while we used the current WHO definition of a modern contraceptive method, we note that women may not encounter the same barriers to using fertility awareness based methods, such as LAM and the standard days methods, as they do for methods procured at a health facility e.g., distance. Given the small number of users in the sample population, we were unable to perform subgroup analyses on users of specific methods or method types. We did not have geolocation data for contraceptive procurement sites other than the PHCs and district referral hospital, and were therefore unable to measure distance to these other locations. Although we assessed relative poverty on contraceptive use, wealth quintiles may be less meaningful in a context where absolute poverty is so widespread. Over three quarters (77.4%) of our sample fell in the poorest wealth quintile relative to a nationally representative sample, and only 5.5% were in Mali’s top two quintiles [2]. Furthermore, small holder wealth in a context like West Africa is difficult to measure as it is accumulated through shifting and diversifying sources (e.g., productive assets, land, labor, remittances, social networks, etc.). Due to seasonality and social desirability bias, we may have underestimated the prevalence of food insecurity. Finally, although we consider the inclusion of empowerment measures a strength of our study on contraceptive use in sub-Saharan Africa, we acknowledge that these measures are “simple windows into complex realities” and thus inherently limited [9]. However, it is likely that our measure of decision-making would underestimate the actual agency women exercise over resources and choice, which may also be exerted through informal or subtle negotiation.

Our multilevel modeling technique allowed us to appropriately model the nested structure of individuals within households within family compounds within communities, and to assess the influences of higher level factors on individual level outcomes. Although women with missing outcome data were different in some observable characteristics, the percentage of missing data was very low (0.4%) and our complete case regression analysis included 95% of women in the sample; therefore, this should not have impacted our results. Finally, by using precise geolocation data at the household and facility levels, our study was able to examine household distance to health center as a predictor of modern contraceptive use and to explore how users were grouped together at the community level. This sets our research apart from much of the multilevel research on the use of reproductive health services that relies on DHS data.

## Conclusion

Women’s decision-making, education, and paid labor force participation, as well as living in a household nearer to a health center and with another women who used modern contraception, were associated with use in this poor access environment. In designing and implementing strategies to expand access to contraception, policymakers and practitioners should consider these axes, sources, and settings for underlying female empowerment. Relevant to the design of accessible and effective community health systems more broadly, our findings suggest that distance to facility is an important barrier to care even within a 5 km radius, and that care available from a fixed community health post on a fee-for-service basis is insufficient to increase utilization.

## Supplementary Information

**Additional File 1:** Household survey instrument

**Additional File 2:** Supplementary tables 1 and 2

## Data Availability

The datasets generated and/or analysed during the current study are not yet publicly available due to the ongoing nature of the cluster-randomized controlled trial, but are available from the corresponding author on reasonable request.

## Abbreviations

AOR: Adjusted odds ratio
CHW: Community health worker
CI: Confidence interval
DHS: Demographic and Health Survey
GIS: Geographic information system
ICC: Intracluster correlation coefficient
IQR: Interquartile Range
IUD: Intrauterine device
LAM: Lactational amenorrhea method
OCP: Oral contraceptive pill
PHC: Primary Health Center
WHO: World Health Organization
