# Supplementary material for: Women’s empowerment, intrahousehold influences, and health system design on modern contraceptive use in rural Mali: a multilevel analysis of cross-sectional survey data
Source: Reprod Health. 2021 Mar 3;18:55. doi: 10.1186/s12978-020-01061-z (PMC7931535; doi:10.1186/s12978-020-01061-z)
Supplement: Supplementary file 1 — Additional File 1: Household survey instrument [file 12978_2020_1061_MOESM1_ESM.docx]

**household survey**

ProCCM Trial: Bankass

**HOUSEHOLD ROSTER:**

1. **LIST OF MEMBERS**

C1. Date

enquete_mois

01-12

enquete_jour

01-31

enquete_an

2016-2017

CensusID. Interviewer ID

INTERVIEWER ID

C2. Language of Interview

6. Other-specify (H103S. Specify (String 100))

5. French

4. Peulh

3. Tomoso

2. Tingu

1. Bamanankan

C3. Health area (NAME)

ANAME (aireS. Specify (String 100))

C4. Village (NAME)

VNAME (villageS. Specify (String 100))

C5. Hamlet (NAME)

N/A

HNAME (hameauS. Specify (String 100))

C6A. Concession number.

- ENTER the concession number, 4 digits

CONCESSIONNO

C6B. Household number.

- ENTER the household number, 2 digits

HOUSEHOLDNUMBER

Household Members Loop (Max = 50)

Identify all household members. A member of the household is someone who usually lives in the household, who shared the meals in the household for at least one year, and who has no other residence. Someone who has recently moved and does not plan to return within a year is not considered a household member.

C7. RECORD Member ID

- Head = 000
- Woman = 100
- BEGIN with the head of the household
- ENTER the member’s identification number

MEMBERID

C8. First & Family Name

NAME (C8S. Specify (String 100))

C16. Gender

2. Female

1. Male

C14. Age

How old is [NAME]?

age_an

Years-Specify (0-100)

age_mois

Months-Specify (0-12)

age_jour

Days-Specify (0-31)

C11. What is [NAME]’s date of birth?

JourNaiss

01-31

MoisNaiss

01-12

AnneeNais

1900-2017

C14CKPT: WHETHER RECORDED DOB MATCHES AGE

|  |  |  |  |
| --- | --- | --- | --- |
|  | 1. RECORDED DATE OF BIRTH DOES NOT MATCH AGE (C14 – C11 ≠ 0) | 1. RECORDED DATE OF BIRTH DOES MATCH AGE (C14 – C11 = 0) | → GO TO C15 |
|  | ↓ |  |  |

***** If the recorded date of birth does not match the age, you must correct the inconsistency. To do this, ask for clarification on age, date of birth, or both. It is important to understand that one or the other of the two pieces of information may be incorrect. Do not assume, for example, that it is always the date of birth that was given correctly and that it is the age that is incorrect. The date or age, or date and age may not be correct.

If the difference between age and DDN is not equal to 0, probe:

C14_2. I have recorded that (NAME) is X months / years, and that he / she was born in (MONTH / YEAR). I want to make sure that I recorded the exact age of (NAME).

(NAME) is how many months / years old?

age_an

Years-Specify (0-100)

age_mois

Months-Specify (0-12)

age_jour

Days-Specify (0-31)

C11_2.

(NAME) was born in what month / year?

AnneeNaiss

1900-2017

MoisNaiss

01-12

JourNaiss

01-31

C15. Relationship to Head of Household

6. Son-in-law / daughter-in-law

5. Brother / sister

4. Father / mother

3. Daughter / son

2. Spouse

1. Head of household

13. Foster child / adopted

12. Visitor

11. Uncle / aunt

10. Nephew / niece

9. Cousin

8. Grandparent

7. Grandchild

14. Other-specify (H115S. Specify (String 100))

C10A. Status of Household Member

- Present = Physically present in the village during the survey period in the village
- Absent/travel = Absent from the village or traveling during the survey period in the village

2. Absent / Travel

1. Present

End of Household Members Loop

**HOUSEHOLD ROSTER:**

1. **MORTALITY**

D1. In the last five years, that is, since the last cereal harvest before ATT's coup d’état until now, has anyone in your household died?

9. No Response

8. Do not Know

2. No

1. Yes

🡪 GO TO 104 🡪 GO TO 104 🡪 GO TO 104

↓

Mortality Loop (Max = 20)

D2. Beginning with the most recent death, please, tell me:

The name of the person?

- ENTER FIRST NAME & FAMILY NAME

NAME (String 100)

FAMILYNAME (String 100)

D3. Relationship to head of household?

6. Son-in-law / daughter-in-law

5. Brother / sister

4. Father / mother

3. Daughter / son

2. Spouse

1. Head of household

13. Foster child / adopted

12. Visitor

11. Uncle / aunt

10. Nephew / niece

9. Cousin

8. Grandparent

7. Grandchild

14. Other-specify (H203S. Specify (String 100))

D4. What is the date of death of [NAME]?

D4_mois

- ENTER BOTH THE MONTH AND YEAR OF DEATH. Both fields are mandatory
- Mark the month in 2 digits, 1 to 12)
- If unknown month, ENTER 88
- If no response, ENTER 99

01-12, 88, 99

D4_annee

- Mark the year in 4 digits, like 1960)
- If unknown year, ENTER 8888.
- If no response, ENTER 9999.

1900-2100, 8888, 9999

D5. What is the date of birth of [NAME]?

D5_jour

- ENTER day
- If unknown day, ENTER 88.
- If no response, ENTER 99.

01-12, 88, 99

D5_mois

- ENTER month
- If unknown month, ENTER 88.
- If no response, ENTER 99.

01-31, 88, 99

D5_annee

- ENTER year
- If unknown month, ENTER 8888.
- If no response, ENTER 9999.

1900-2100, 8888, 9999

D6. How old was (NAME) when he died?

- ALL FIELDS ARE MANDATORY
- ENTER years of age
- MARK 00 years if the child is under one year
- If unknown number of years, ENTER 88
- If no response, ENTER 99

00-99

- ENTER months of age
- MARK 00 months if the child is less than one month old.
- If unknown number of months, ENTER 88
- If no response, ENTER 99

00-99

- ENTER days of age
- If unknown number of days, ENTER 88.
- If no response for age, ENTER 99.

00-99

D7CKPT: WHETHER RECORDED DOB MATCHES AGE

|  |  |  |  |
| --- | --- | --- | --- |
|  | 1. RECORDED DATE OF DEATH DOES NOT MATCH AGE (D5–D6 ≠ 0) | 1. RECORDED DATE OF DEATH DOES MATCH AGE (D5–D6=0) | → GO TO D8 |
|  | ↓ |  |  |

If the difference between the age of death and the date of death minus the date of birth is not 0, ask questions 7B to 7D. If the difference is 0, go to Q8:

D7B. I recorded that (NAME) was X months / years, and that he / she died in (MONTH / YEAR). I want to make sure that I recorded the exact age of (NAME) at the time of death. How old was (NAME) when he died?

- ENTER years
- If unknown years, ENTER 88
- If no response, enter 99

0-99

- ENTER months
- If unknown months, ENTER 88
- If no response, ENTER 99

0-99

- ENTER days
- If unknown days, ENTER 88
- If no response, ENTER 99

0-99

D7C. I want to make sure that I have entered the date of death of (NAME). What is the date of death of (NAME)?

- ENTER month (mark the month in 2 digits, 1 to 12)
- If unknown month, ENTER 88
- If no response, ENTER 99

01-12, 88, 99

- ENTER year (mark the year in 4 digits, like 1960)
- If unknown year, ENTER 8888
- If no response, ENTER 9999

1900-2100, 8888, 9999

D7D. I want to make sure that I have entered the date of birth of (NAME). What is the date of birth of (NAME)?

- ENTER day
- If unknown day, ENTER 88
- If no response, ENTER 99

01-31, 88, 99

- ENTER month
- If unknown month, ENTER 88
- If no response, ENTER 99

01-12, 88, 99

- ENTER year
- If unknown year, ENTER 8888
- If no response, ENTER 9999

1900-2100, 8888, 9999

D8. If child, is the child's biological mother currently living in the household?

9. No Response

8. Do not Know

1. Yes

2. No

 🡪 GO TO H210 🡪 GO TO H210 🡪 GO TO H210

↓

MereID. Mark the last three digits of the mother’s ID.

000-999

D10. Is the biological father of the child currently living in the household?

9. No Response

8. Do not Know

2. No

1. Yes

🡪 GO TO H212 🡪 GO TO H212 🡪 GO TO H212

↓

PereID. Mark the father’s ID.

000-999

D12. Was (NAME) male or female?

2. Female

1. Male

🡪 GO TO H216

↓

D13. Was she pregnant when she died?

9. No Response

8. Do not Know

2. No

1. Yes

D14. Did she die during childbirth?

9. No Response

8. Do not Know

2. No

1. Yes

🡪 GO TO H216

↓ ↓ ↓

D15. Did she die within two months of giving birth or ending the pregnancy (abortion)?

9. No Response

2. No

8. Do not Know

1. Yes

D16. In the last five years, that is, from the last cereal harvest before the ATT coup d’état to [DATE], did anyone else who lived in your household die?

9. No Response

8. Do not Know

2. No

1. Yes

🡪 GO TO H202

↓ ↓ ↓

End of Mortality Loop (Max = 20)

104. How many people does your household currently have?

- This is the household and not the concession. Count those who usually live in your household
- If the number of people in the household is unknown, ENTER 88.
- If there is no response, ENTER 99.

0-99

**HOUSEHOLD ROSTER:**

**(3) HOUSEHOLD CHARACTERISTICS**

116A. What is the main source of drinking water for members of your household?

- If piped water, SPECIFY:

14. Public tap / standpipe

13. Piped to neighbor

12. Piped into yard/plot

11. Piped into dwelling

21. Tube well or borehole

- If dug well, SPECIFY:

31. Protected well

32. Unprotected well

- If water from spring, SPECIFY:

42. Unprotected spring

41. Protected spring

- If other, SPECIFY:
- NOTE: Surface water could be river, dam, lake, pond, stream, canal, irrigation channel

91. Bottled water

81. Surface water

71. Cart with small tank

61. Tanker truck

51. Rainwater

99. No response

88. Do not know

96. Other-specify (116AS. Specify (String 100))

116B. Do you do anything to the water to make it safer to drink?

9. No Response

8. Do not Know

2. No

1. Yes

🡪 GO TO 117A 🡪 GO TO 117A 🡪 GO TO 117A

↓

116C. What do you usually do to make the water safer to drink?

- ENTER all that apply
- PROBE: Anything else?

4. Use water filter (ceramic/sand/composite/etc)

3. Strain through a cloth

2. Add bleach/chlorine

1. Boil

88. Do not Know

7. Other-specify (116CS. Specify (String 100))

6. Let it stand and settle

5. Solar disinfection

99. No Response

116D. How often do you treat water to drink?

99. No Response

88. Do not Know

4. Rarely

3. Sometimes

2. Often

1. Always

117A. What kind of toilet facility do members of your household usually use?

- If flush or pour flush toilet, SPECIFY:

14. Flush to somewhere else

13. Flush to pit latrine

12. Flush to septic tank

11. Flush to piped sewer system

15. Flush, don’t know where

- If pit latrine, SPECIFY:

23. Pit latrine without slab/open pit

22. Pit latrine with slab

21. Ventilated improved pit latrine

- If other, SPECIFY:

61. No facility/bush/field

51. Hanging toilet

41. Bucket toilet

31. Composting toilet

99. No response

88. Do not Know

96. Other-specify (117AS. Specify (String 100))

117B. Where is this toilet facility located?

3. Elsewhere

2. In own yard/plot

1. In own dwelling

117C. Do you share this toilet facility with other households?

2. No

1. Yes

118. Does your household have:

- Electricity?

2. No

1. Yes

- A radio?

2. No

1. Yes

- A television ?

2. No

1. Yes

- A mobile phone ?

2. No

1. Yes

- A non-mobile phone?

2. No

1. Yes

- A refrigerator ?

2. No

1. Yes

- An antenna TV5?

2. No

1. Yes

- Subscription to Cable?

2. No

1. Yes

- A washing machine?

2. No

1. Yes

- A stove or a gas or electric cooker?

2. No

1. Yes

- An improved home?

2. No

1. Yes

- A video / CD / DVD player?

2. No

1. Yes

- An air conditioner?

2. No

1. Yes

- A computer?

2. No

1. Yes

- Internet at home?

2. No

1. Yes

119. What type of fuel does your household mainly use for cooking?

08. Wood

07. Charcoal

06. Coal, lignite

05. Kerosene

04. Biogas

03. Natural Gas

02. LPG

01. Electricity

95. No food cooked in household

11. Animal Dung

10. Agricultural crop

09. Straw / shrubs / grass

99. No Response

88. Do not Know

96. Other-specify (119S. Specify (String 100))

120. OBSEVE MAIN MATERIAL OF THE FLOOR OF THE DWELLING. RECORD OBSERVATION.

- If natural floor, SPECIFY:

12. Dung

11. Earth/Sand

- If rudimentary floor, SPECIFY:

22. Palms / bamboo

21. Wooden planks

- If finished floor, SPECIFY:

35. Carpet

34. Cement

33. Ceramic tiles

31. Parquet or polished wood

32. Vinyl or asphalt strips

96. Other-specify (120S. Specify (String 100))

121. Does any member of this household own:

- A watch ?

2. No

1. Yes

- A plow?

2. No

1. Yes

- A bicycle ?

2. No

1. Yes

- A motorcycle or a scooter?

2. No

1. Yes

- An animal-drawn cart?

2. No

1. Yes

- A canoe / or fishing nets?

2. No

1. Yes

- A tractor ?

2. No

1. Yes

- A car or truck?

2. No

1. Yes

- A boat with a motor ?

2. No

1. Yes

122. How many of the following animals does this household own?

- NONE = 00
- 95 OR MORE = 95
- DO NOT KNOW = 88
- NO RESPONSE = 99
- Livestock?

00-99

- Dairy cows or bulls?

00-99

- Horses, donkeys or mules?

00-99

- Goats?

00-99

- Sheep?

00-99

- Chickens?

00-99

123. In this household, how many rooms / places are used for sleeping?

- ENTER AND SPECIFY ALL THAT APPLY:

Places (ENTER 00-99)

Rooms (ENTER 00-99)

124. In the last 30 days, was there ever no food to eat of any kind in your house because of a lack of resources to get food?

9. No Response

8. Do not Know

2. No

1. Yes

🡪 GO TO 126 🡪 GO TO 126 🡪 GO TO 126

↓

125. How often did this happen in the last 30 days?

2. A few times (3-10 times)

1. Rarely (1-2 times)

3. Often (more than 10 times)

126. In the past 30 days, did you or any household member go to sleep at night hungry because there was not enough food?

8. Do not Know

2. No

1. Yes

🡪 GO TO 128 🡪 GO TO 128

↓

127. How often did this happen in the last 30 days?

3. Often (more than 10 times)

2. A few times (3-10 times)

1. Rarely (1-2 times)

128. In the past 30 days, did you or any household member go a whole day and night without eating anything because there was not enough food?

8. Do not Know

2. No

1. Yes

🡪 GO TO 130 🡪 GO TO 130

↓

129. How often did this happen in the last 30 days?

3. Often (more than 10 times)

2. A few times (3-10 times)

1. Rarely (1-2 times)

**WOMEN’S SURVEY SCREENING**

**IDENTIFICATION OF THE WOMAN INTERVIEWED**

C1. Date

enquete_mois

01-12

enquete_jour

01-31

enquete_an

2016-2017

C2. ENTER name and surname of the interviewer:

INTNAME (C2S. Specify (String 100))

C3. Interviewer number:

- ENTER interviewer number

INTERVIEWER NUMBER

C4. Name of the village

VNAME (C4S. Specify (String 100))

C5. Name and surname of the head of the family

CHEFNAME (C5S. Specify (String 100))

C6. Telephone number of the head of the family

- ENTER the telephone number of the head of the family

CHEFPHONENO

C7. Name and surname of the woman

NAME (C7S. Specify (String 100))

C8. Age (in years) of woman

00-99

C9. Name and surname of the woman’s husband

HUSNAME (C9S. Specify (String 100))

C10. Telephone number of woman’s husband

- ENTER the telephone number of the woman’s husband

HUSPHONENO

**INFORMATION ON THE SCREENING OF THE WOMAN INTERVIEWED**

E1. Is the woman aged 15-49 years?

2. No

1. Yes

E2. Permanent resident?

- NB: A permanent resident is someone who usually lives in the household, who shared meals in the household for at least one year, and who has no other residence. Someone who has recently moved and does not plan to return within the year is not considered a permanent resident

1. Yes

2. No

E3. Is the woman expected to remain in the study area during the next three years?

2. No

1. Yes

E4. Is the woman willing to participate and complete the study program?

2. No

1. Yes

E5CKPT. ELIG CHECKPOINT: WHETHER PARTICIPANT IS ELIGIBLE OR NOT

- If No to one of questions 1, 2, 3 and 4 the woman is not eligible for study. So do not continue the interview
- If yes to all questions 1, 2, 3 and 4 then the woman is eligible

🡪GO TO E8

2. Not Eligible

1. Yes, Eligible

E6. Is the woman eligible?

2. No

1. Yes

E7. If eligible, is she included in the study?

2. No

1. Yes

🡪GO TO E9

E8. If not included, give reasons:

- 1. Declined consent

2. No

1. Yes

- 1. Need to consult family

2. No

1. Yes

- 1. Not convinced

2. No

1. Yes

- 1. Family refused consent

2. No

1. Yes

🡪END OF INTERVIEW

E9. Randomized village

2. I-CCM

1. Pro-CCM

**Pro-CCM Randomized Controlled Trial Survey Tool**

V10112016

**Section 1: Demographic Details**

101. Have you ever attended school (madrasah or French)?

9. No response

8. Do not Know

2. No

1. Yes

🡪 GO TO 103 🡪 GO TO 103 🡪 GO TO 103

↓

102. What is the highest level of school you have completed:

- Primary 1 (1 cycle), primary 2 (2nd cycle), secondary (high school, technical) or higher?

4. More than secondary

3. Secondary (high school / technical)

2. Primary 2 (2^nd^ cycle)

1. Primary 1 (1^st^ cycle)

9. No Response

8. Do not Know

103. Now I would like you to read this sentence to me.

- SHOW CARD TO RESPONDENT.
- IF THE RESPONDENT CANNOT READ WHOLE SENTENCE, PROBE: Can you read any part of the sentence to me?
- If no card with required language, SPECIFY which language.

3. Able to read whole sentence

2. Able to read only part of the sentence

1. Cannot read at all

5. Blind / visually impaired

4. No card with required language-specify (103S. Specify (String 100))

105. What religion do you practice?

6. Animist

5. Other Christian religion

4. Evangelical

3. Methodist

2. Catholic

1. Muslim

99. No Response

88. Do not Know

8. Without Religion

7. Other religions-specify (105S. Specify (String 100))

106. What is your ethnicity?

8. Tamachek / Bella

7. Sonraï

6. Sarakole / soninke / marka

5. Malinke

4. Mossi

3. Bambara

2. Peulh

1. Dogon

13. CEDEAO country

12. Other Malian ethnic-specify (106S. Specify (String 100))

11. Boso

10. Bobo

9. Sénoufo / Minianka

15. Other non-African countries-specify (106S. Specify (String 100))

14. Other African countries-specify (106S. Specify (String 100))

99. No response

88. Do not know

107. Are you currently married or living together with a man as if married?

2. Yes, living with a man

1. Yes, currently married

🡪 GO TO 110 🡪GO TO 110

9. No Response

8. Do not Know

3. No, not in union

↓ ↓ ↓

108. Have you ever been married or lived together with a man as if married?

3. No

2. Yes, lived with a man

1. Yes, formerly married

🡪GO TO 130

↓ ↓

109. What is your marital status now: are you widowed, divorced or separated?

1. Widowed

3. Separated

2. Divorced

🡪GO TO 114 🡪GO TO 114 🡪GO TO 114

110. Is your (husband/partner) living with you now or is he staying elsewhere?

3. Staying elsewhere

2. Living with her sometimes

1. Living with her

🡪GO TO 111 🡪GO TO 111

↓

9. No Response

8. Do not Know

↓ ↓

110B. HUSBAND/PARTNER [YES, MARRIED/YES, LIVING WITH]

During the month, how much time does your [husband / partner] live with you in this household?

3. 75%

2. 50%

1. 25%

110C. Where is his other home?

2. In another village-specify (110CS. Specify (String 100))

1. In this village

111. Does your (husband / partner) have other wives or does he live with other women as if married?

9. No Response

8. Do not Know

2. No

1. Yes

🡪GO TO 114

↓ ↓ ↓

112. Including yourself, in total, how many wives or live-in partners does he have?

- ENTER total number of wives and live-in partners
- If unknown, ENTER “88”
- If no response, ENTER “99”

00-99

113. Are you the first, second, ........ wife?

- ENTER rank
- If rank unknown, ENTER “88”
- If no response, ENTER “99”

00-99

114. What is the highest level of school your (husband/partner) has completed:

- Primary 1 (1 cycle), primary 2 (2nd cycle), secondary (high school, technical) or higher?

1. Primary 1 (1^st^ cycle)

4. More than secondary

3. Secondary (high school / technical)

2. Primary 2 (2^nd^ cycle)

99. No Response

88. Do not Know

115. What is your (husband’s/partner’s) occupation? That is, what kind of work does he mainly do?

7. Contracted

6. Official

5. Bricklayer

4. Butcher

3. Trader

2. Breeder

1. Cultivator

99. No Response

88. Do not Know

8. Other-specify (115S. Specify (String 100))

130INTRO. As you know, some women take up jobs for which they are paid in cash or kind. Others sell things, have a small business or work on the family farm or in the family business.

- ENTER [1] to continue

1. Continue

130. During the past month, did you do any of these things or any other work for which you were paid in cash or in kind?

9. No Response

8. Do not Know

2. No

1. Yes

131. What is your occupation? That is, what kind of work do you mainly do?

7. Other-specify (131S. Specify (String 100))

6. Contracted

5. Official

4. Planter

3. Trader

2. Small business

1. Housewife

99. No Response

88. Do not Know

132. Do you do this work for a member of your family, for someone else, or are you self-employed?

3. Self-employed

9. No Response

8. Do not Know

2. For someone else

1. For family member

133. Do you usually work throughout the year, or do you work seasonally, or only once in a while?

9. No Response

8. Do not Know

3. Once in a while

2. Seasonally / part of the year

1. Throughout the year

134A. Are you paid in cash or kind for this work or are you not paid at all?

4. Not paid

3. In kind only

2. Cash and kind

1. Cash only

🡪GO TO 134B 🡪GO TO 134B 🡪GO TO 134C 🡪GO TO 136

9. No Response

8. Do not Know

🡪GO TO 135 🡪GO TO 135

134B. How much money do you get for this job?

- - ENTER the value in FCFA:
  - If value unknown, ENTER “8888”
  - If no response, ENTER “9999”

0000-9999

134BCKPT.

🡪GO TO 134D

↓

1. If 134A=1

2. If 134A=2 OR 134A=3

134C. How much, in your estimation, is the cash value of this payment in kind?

- - ENTER the value in FCFA:
  - If value unknown, ENTER “8888”
  - If no response, ENTER “9999”

0000-9999

134D. How often are you paid?

9. No Response

8. Do not Know

4. Other-specify (134DS. Specify (String 100))

3. Monthly

2. Weekly

1. Daily

135. Who usually decides how the money you earn will be used?

5. Respondent and concession leader jointly

4. Respondent and husband/partner jointly

3. Concession leader

2. Husband/Partner

1. Respondent

99. No Response

88. Do not Know

7. Other-specify (135S. Specify (String 100))

6. Husband and concession leader jointly

136. HUSBAND/PARTNER [YES, MARRIED/YES, LIVING WITH]

Who usually decides how your (husband’s/partner’s) earnings will be used?

5. Respondent and concession leader jointly

4. Respondent and husband/partner jointly

3. Concession leader

2. Husband/Partner

1. Respondent

99. No Response

88. Do not Know

7. Other-specify (136S. Specify (String 100))

6. Husband and concession leader jointly

137. Who usually makes decisions about health care for yourself?

5. Respondent and concession leader jointly

4. Respondent and husband/partner jointly

3. Concession leader

2. Husband/Partner

1. Respondent

99. No Response

88. Do not Know

7. Other-specify (137S. Specify (String 100))

6. Husband and concession leader jointly

138. Who usually makes decisions about making major household purchases?

5. Respondent and concession leader jointly

4. Respondent and husband/partner jointly

3. Concession leader

2. Husband/Partner

1. Respondent

99. No Response

88. Do not Know

7. Other-specify (138S. Specify (String 100))

6. Husband and concession leader jointly

139. Who usually makes decisions about visits to your family or relatives?

5. Respondent and concession leader jointly

4. Respondent and husband/partner jointly

3. Concession leader

2. Husband/Partner

1. Respondent

99. No Response

88. Do not Know

7. Other-specify (139S. Specify (String 100))

6. Husband and concession leader jointly

140. Sometimes a husband is upset or irritated by the things his wife does. In your opinion, is a husband justified in hitting or beating his wife in the following situations:

[A] If she goes out without telling him?

2. No

8. Do not Know

1. Yes

9. No Response

[B] If she neglects the children?

2. No

8. Do not Know

1. Yes

9. No Response

[C] If she argues with him?

2. No

8. Do not Know

1. Yes

9. No Response

[D] If she refuses to have sex with him?

9. No Response

8. Do not Know

2. No

1. Yes

[E] If she burns the food?

2. No

1. Yes

9. No Response

8. Do not Know

[F] If she uses contraceptives without the husband's consent?

8. Do not Know

2. No

1. Yes

9. No Response

[G] If she argues with the husband / partner's parents?

9. No Response

8. Do not Know

2. No

1. Yes

141. Have you ever:

- Been to the market?

1. Yes

8. Do not Know

2. No

9. No Response

- Been there alone?

8. Do not Know

2. No

1. Yes

9. No Response

- Been to the hospital/clinic/doctor?

2. No

8. Do not Know

1. Yes

9. No Response

- Gone there alone?

9. No Response

2. No

1. Yes

8. Do not Know

- Gone to the cinema?

8. Do not Know

2. No

1. Yes

9. No Response

- Gone there alone?

8. Do not Know

2. No

1. Yes

9. No Response

- Gone outside the village?

9. No Response

2. No

1. Yes

8. Do not Know

- Gone there alone?

9. No Response

8. Do not Know

2. No

1. Yes

**Section 1B: CHW Care**

142. Do you know how to contact or find your Community Health Worker in case of need?

3. I have no CHW

2. No

1. Yes

9. No Response

8. Do not Know

143. During the past month, have you met your community health worker for advice or care?

9. No Response

8. Do not Know

2. No

1. Yes

🡪GO TO 201 🡪GO TO 201 🡪GO TO 201

↓

144. If so, where did you meet the CHW?

9. No Response

8. Do not Know

3. Other-specify (144S. Specify (String 100))

2. At the CHW site

1. At your house

145. How many times?

- - ENTER number of times
  - If unknown number of times, ENTER “88”
  - If no response, ENTER “99”

00-99

**Section 2: Reproductive health**

**2A.** **Family planning**

201. Are you or your partner currently doing something or using any method to delay or avoid getting pregnant?

9. No Response

8. Do not Know

2. No

1. Yes

🡪GO TO 218 🡪GO TO 218 🡪GO TO 218

↓

202. What method are you using?

- RECORD ALL MENTIONED.

8. Female Condom

7. Condom

6. Pill

5. Injectables

4. Implants

2. Male Sterilization

3. IUD

1. Female Sterilization

12. LAM (Lactational Amenorrhea Method)

11. Necklace (Standard Days Method)

10. Foam / Jelly / Spermicidal Tablet

9. Diaphragm

99. No Response

88. Do not Know

15. Other method-specify (202S. Specify (String 100))

14. Withdrawal

13. Rhythm Method

203. [CURRENT METHOD] = RESPONSE TO 202

Since what month and year have you been using [CURRENT METHOD] without stopping?

- Instruct the participant to estimate if she does not recall the exact date
- RECORD month
- If unknown, ENTER “88”
- If no response, ENTER “99”

01-12, 88, 99

- ENTER year
- If unknown, ENTER “8888”
- If no response, ENTER “9999”

1900-2100, 8888, 9999

204A. [CURRENT METHOD] = RESPONSE TO 202, [DATE 203] = RESPONSE TO 203

You started using [CURRENT METHOD] in [DATE 203]. Where did you get it at that time?

- If primary health center, SPECIFY which primary health center

3. Regional Hospital

2. National Hospital

1. At home

🡪GO TO 205 🡪GO TO 205 🡪GO TO 205

5. Primary health center-specify (204AS. Specify (String 100))

4. Community Referral Hospital (Bankass)

🡪GO TO 205 🡪GO TO 205

🡪GO TO 204B 🡪GO TO 205 🡪GO TO 205

8. Shop

7. Hospital / Private Clinic

6. Community Health Worker

11. Acquaintance / Friend / Parent

10. Street Vendor

9. Nightclub / kiosk / bar

🡪GO TO 205 🡪GO TO 205 🡪GO TO 205

99. No Response

12. Other-specify (204AS. Specify (String 100))

88. Do not Know

🡪GO TO 205 🡪GO TO 205 🡪GO TO 205

204B. Where did you meet the CHW to get the method at that time?

9. No Response

8. Do not Know

3. Other-specify (204BS. Specify (String 100))

2. CHW Site
HW

1. At respondent’s house

205. Has anyone referred you to this place for family planning?

9. No Response

8. Do not Know

2. No

1. Yes

🡪GO TO 207

↓ ↓ ↓

206. If so, who referred you to this place?

- - RECORD ALL MENTIONED

5. A health worker from primary health center

4. Another member of the family

3. Husband

2. A friend

1. CHW

99. No Response

88. Do not Know

6. Other-specify (206S. Specify (String 100))

207. [CURRENT METHOD] = RESPONSE TO 202

Who advised you to use [CURRENT METHOD] family planning?

- - RECORD ALL MENTIONED

4. Health worker at primary health center

3. CHW

2. Husband

1. Respondent

5. Nightclub / kiosk / bar

88. Do not Know

8. Other-specify (207S. Specify (String 100))

7. Acquaintance / friend / parent

6. Street vendor

99. No Response

208A. [CURRENT METHOD] = RESPONSE TO 202

Where did you get [CURRENT METHOD] last time?

- - If primary health center, SPECIFY which primary health center

1. At home

3. Regional Hospital

2. National Hospital

🡪GO TO 209 🡪GO TO 209 🡪GO TO 209

4. Community Referral Hospital (Bankass)

🡪GO TO 209

5. Primary health center-specify (208AS. Specify (String 100))

🡪GO TO 209

8. Shop

7. Hospital / Private Clinic

6. Community Health Worker

🡪GO TO 208B 🡪GO TO 209 🡪GO TO 209

10. Street Vendor

9. Nightclub / kiosk / bar

🡪GO TO 209 🡪GO TO 209

11. Acquaintance / Friend / Parent

🡪GO TO 209

88. Do not Know

12. Other-specify (208AS. Specify (String 100))

🡪GO TO 209 🡪GO TO 209

99. No Response

🡪GO TO 209

208B. Where did you meet the CHW to get the method last time?

8. Do not Know

3. Other-specify (208BS. Specify (String 100))

2. CHW Site
HW

1. At respondent’s house

🡪GO TO 214

↓ ↓ ↓

9. No Response

↓

209. [CURRENT METHOD] = RESPONSE TO 202

In total, how much did you pay to get to the health center where you got [CURRENT METHOD] the last time?

- - ENTER the value in silver
  - If not paid, ENTER “7777”
  - If unknown, ENTER “8888”
  - If no response, ENTER “9999”

0000-9999

210. [CURRENT METHOD] = RESPONSE TO 202

Approximately how long it takes to get to the health center where you got [CURRENT METHOD] the last time?

2. 1 hour (time between fitri and saafo)

1. Less than an hour (less than the time between fitri and saafo)

99. No Response

88. Do not Know

6. 2 or more days

5. 1 day

4. ½ day

3. 2 hours (time between salifana and star)

211. How long does the time it takes to get to the health center seem to you?

99. No Response

88. Do not Know

5. Very short

4. Short

3. Normal

2. Long

1. Very long

212. Once at the health center, how long did you wait before receiving care?

2. 1 hour (time between fitri and saafo)

1. Less than an hour (less than the time between fitri and saafo)

99. No Response

88. Do not Know

6. 2 or more days

5. 1 day

4. ½ day

3. 2 hours (time between salifana and star)

213. How long does the waiting time seem to you?

99. No Response

88. Do not Know

5. Very short

4. Short

3. Normal

2. Long

1. Very long

214. How long does the family planning consultation take?

2. 1 hour (time between fitri and saafo)

1. Less than an hour (less than the time between fitri and saafo)

3. 2 hours (time between salifana and star)

99. No Response

88. Do not Know

6. 2 or more days

5. 1 day

4. ½ day

215. How much did you pay for the family planning method?

- ENTER value in silver:
- If not paid, ENTER “7777”
- If unknown, ENTER “8888”
- If no response, ENTER “9999”

0000-9999

216. In addition to what you told me, do you have other expenses related to this family planning method?

9. No Response

8. Do not Know

2. No

1. Yes

🡪GO TO 218 / 🡪GO TO 218 🡪GO TO 218

↓

217. How much have you spent on: ?

A. Other medications

- ENTER value
- If unknown, ENTER “8888”
- If no response, ENTER “9999”

0000-9999

B. Food and accommodations

- ENTER value
- If unknown, ENTER “8888”
- If no response, ENTER “9999”

0000-9999

C. Other expenses

- ENTER value
- If unknown, ENTER “8888”
- If no response, ENTER “9999”

0000-9999

218. Over the last 12 months, were you interested in learning the methods of family planning?

9. No Response

8. Do not Know

2. No

1. Yes

219. Have you consulted with someone to learn more?

9. No Response

8. Do not Know

2. No

1. Yes

🡪GO TO 221 🡪GO TO 221 🡪GO TO 221

↓

220A. Who did you consult with to learn more?

3. CHW

2. Husband

1. Respondent

🡪GO TO 221 🡪GO TO 221 🡪GO TO 220B

5. Nightclub / kiosk / bar

4. Health worker at primary health center

🡪GO TO 221 🡪GO TO 221

7. Acquaintance / friend / parent

6. Street vendor

🡪 GO TO 221 🡪GO TO 221

99. No Response

88. Do not Know

8. Other-specify (220AS. Specify (String 100))

🡪GO TO 221 🡪GO TO 221 🡪GO TO 221

220B. Where did you consult with the CHW?

9. No Response

8. Do not Know

3. Other-specify (220BS. Specify (String 100))

2. CHW Site
HW

1. At respondent’s house

221. Over the past 12 months, did you receive a visit from a community health worker who told you about family planning?

9. No Response

8. Do not Know

2. No

1. Yes

🡪GO TO 228 🡪GO TO 228 🡪GO TO 228

↓

222. When was the last visit of the community health worker to talk about family planning?

- ENTER Value

0-50

- ENTER Unit

2. Weeks

3. Months

1. Days

223. What type of health worker visited you to talk about family planning.

- RECORD ALL MENTIONED

6. Traditional birth attendant

5. CHW

4. Matron

3. Midwife

2. Nurse

1. Doctor

99. No Response

88. Do not Know

7. Other-specify (223S. Specify (String 100))

224. Did the health worker tell you about other family planning methods that you could use?

9. No Response

8. Do not Know

2. No

1. Yes

225. During the family planning consultation, did the health worker offer you a pregnancy test?

9. No Response

8. Do not Know

2. No

1. Yes

🡪GO TO 227 🡪GO TO 227 🡪GO TO 227

↓

226. If you have had a negative pregnancy test, did the health worker propose family planning?

9. No Response

8. Do not Know

3. I had a positive pregnancy test

2. No

1. Yes

227. Were you satisfied with the family planning services delivered by the health worker?

99. No Response

88. Do not Know

3. Satisfied

2. Neutral

1. Not satisfied

**2B. Pregnancy and Childbirth**

228. Are you pregnant now?

9. No Response

8. Do not Know

2. No

1. Yes

🡪GO TO 230 🡪GO TO 230 🡪GO TO 230

↓

229. How many months pregnant are you?

- RECORD NUMBER OF COMPLETED MONTHS.
- If unknown, ENTER “88”
- If no response, ENTER “99”

0-9, 88, 99

230. When did your last menstrual period start?

- Encourage the participant to make her best estimate if she does not know the exact number.
- If menopause or hysterectomy, ENTER “94”
- If before last birth, ENTER “95”
- If never menstruated, ENTER “96”
- If unknown, ENTER “88”
- If no response, ENTER “99”
- ENTER value

0-99

- ENTER unit

3. Months

2. Weeks

1. Days

231. What name was given to your last child?

- ENTER name of child

NAME (String 100)

232INTRO. [NAME] = RESPONSE TO 231

Now I want to ask you questions about your pregnancy with [NAME]

- ENTER [1] to continue

1. Continue

232. Did you have a test to confirm the pregnancy?

9. No Response

8. Do not Know

2. No

1. Yes

🡪GO TO 236 🡪GO TO 236 🡪GO TO 236

↓

233. Who gave you the first test to confirm pregnancy?

6. Traditional birth attendant

4. Matron

5. CHW

3. Midwife

2. Nurse

1. Doctor

99. No Response

88. Do not Know

7. Other-specify (233S. Specify (String 100))

234. Where did you do your pregnancy test?

3. Other-specify (234S. Specify (String 100))

2. In a health center

1. At home

235. How much did you pay for your pregnancy test?

- ENTER value in FCFA
- If unknown, ENTER “8888”
- If no response, ENTER “9999”

0-9999

236. Did you see anyone for antenatal care for this pregnancy?

9. No Response

8. Do not Know

2. No

1. Yes

🡪GO TO 243 🡪GO TO 243 🡪GO TO 243

↓

237. Whom did you see?

- - Anyone else?
  - PROBE TO IDENTIFY EACH TYPE OF PERSON AND RECORD ALL MENTIONED.

6. Traditional birth attendant

5. CHW

4. Matron

3. Midwife

2. Nurse

1. Doctor

99. No Response

88. Do not Know

7. Other-specify (237S. Specify (String 100))

238. Where did you receive antenatal care for this pregnancy?

- Anywhere else?
- PROBE TO IDENTIFY THE TYPE OF SOURCE.

5. District Hospital

4. Regional Hospital

3. National Hospital

1. Your home

2. Other house

8. Pharmacy

7. Clinic / Private Hospital

6. Primary health center-specify (238S. Specify (String 100))

9. Other-specify (238S. Specify (String 100))

99. No Response

88. Do not Know

239A. Who referred you to get antenatal care?

3. CHW

2. Husband

1. Respondent

🡪GO TO 240 🡪GO TO 240 🡪GO TO 239B

5. Nightclub / kiosk / bar

4. Health worker at primary health center

🡪GO TO 240 🡪GO TO 240

7. Acquaintance / friend / parent

6. Street vendor

🡪GO TO 240 🡪GO TO 240

8. Other-specify (239AS. Specify (String 100))

99. No Response

88. Do not Know

🡪GO TO 240 🡪GO TO 240 🡪GO TO 240

239B. Where was the reference by the CHW made?

9. No Response

8. Do not Know

3. Other-specify (239BS. Specify (String 100))

2. CHW Site
HW

1. At respondent’s house

240. How many months pregnant were you when you first received antenatal care for this pregnancy?

- ENTER value in months
- If unknown, ENTER “88”
- If no response, ENTER “99”

0-9, 88, 99

241. How many times did you receive antenatal care during this pregnancy?

- ENTER value
- If unknown, ENTER “88”
- If no response, ENTER “99”

0-99

242. During one of these prenatal visits, were you told about danger signs, warning signs, or complications in pregnancy?

9. No Response

8. Do not Know

2. No

1. Yes

243. During this pregnancy, did you take medicine to prevent malaria?

9. No Response

8. Do not Know

2. No

1. Yes

🡪GO TO 247

↓ ↓ ↓

244. What medications did you take?

- RECORD ALL MENTIONED.
- IF TYPE OF MEDICINE IS NOT DETERMINED, SHOW ALL CURRENT ANTIMALARIAL IN THE SURVEY TO RESPONDENT.

99. No Response

88. Do not Know

3. Other-specify (244S. Specify (String 100))

2. Chloroquine

1. SP / Fansidar

🡪GO TO 247

↓

245. How many times did you take SP/Fansidar during this pregnancy?

- ENTER number of times
- If unknown, ENTER “88”
- If no response, ENTER “99”

0-99

246. Did you get the SP / Fansidar under supervision of medical staff at the health center?

9. No Response

8. Do not Know

2. No

1. Yes

247. [NAME] = RESPONSE TO 231

Who assisted with the delivery of [NAME]?

- Anyone else ?
- PROBE FOR THE TYPE(S) OF PERSON(S) AND RECORD ALL MENTIONED.
- IF RESPONDENT SAYS NO ONE ASSISTED, PROBE TO DETERMINE WHETHER ANY ADULTS WERE PRESENT AT THE DELIVERY.

8. Respondent (alone)

7. Friend/Parent

6. Traditional birth attendant

5. CHW

4. Matron

3. Midwife

2. Nurse

1. Doctor

99. No Response

88. Do not Know

9. Other-specify (247S. Specify (String 100))

248. [NAME] = RESPONSE TO 231

Where did you give birth to [NAME]?

- PROBE TO IDENTIFY THE TYPE OF SOURCE.
- IF UNABLE TO DETERMINE IF PUBLIC OR PRIVATE SECTOR, WRITE THENAME OF THE PLACE

NAME OF PLACE (String 100)

- If at home, SPECIFY

2. Other home

1. Her home

- If public sector, SPECIFY

6. Dispenp / maternity

5. Community referral hospital (Bankass)

4. Regional hospital

3. National hospital

8. Other public sector

7. Primary Health Center

- If private medical center, SPECIFY

13. Other private sector

12. Pharmacy

11. Treatment Room

10. Office of private care

9. Private Clinic / Hospital

- If other, SPECIFY

99. No Response

88. Do not Know

15. Other-specify (248S. Specify (String 100))

14. Transit

249A. Who referred you to this place for the birth?

- RECORD ALL MENTIONED

3. CHW

2. Husband

1. Respondent

🡪GO TO 250 🡪GO TO 250 🡪GO TO 249B

5. Nightclub / kiosk / bar

4. Health worker at primary health center

🡪GO TO 250 🡪GO TO 250

7. Acquaintance / friend / parent

6. Street vendor

🡪 GO TO 250 🡪GO TO 250

99. No Response

88. Do not Know

8. Other-specify (249AS. Specify (String 100))

🡪GO TO 250 🡪GO TO 250 🡪GO TO 250

249B. Where was the reference by the CHW made?

8. Do not Know

9. No Response

3. Other-specify (249BS. Specify (String 100))

2. CHW Site
HW

1. At respondent’s house

250. [NAME] = RESPONSE TO 231

How long after (NAME) was delivered did you stay there?

- ENTER value
- If unknown, ENTER “88”
- If no response, ENTER “99”

0-99

- ENTER unit

3. Weeks

2. Days

1. Hours

251. [NAME] = RESPONSE TO 231

Was (NAME) delivered by caesarean, that is, did they cut your belly open to take the baby out?

9. No Response

8. Do not Know

2. No

1. Yes

252. I would like to talk to you about checks on your health after delivery, for example, someone asking you questions about your health or examining you. Did anyone check on your health while you were still in the facility?

9. No Response

8. Do not Know

2. No

1. Yes

🡪GO TO 256

↓ ↓ ↓

253. Who checked on your health at that time?

- RECORD ALL MENTIONED
- PROBE for most qualified person

6. Traditional birth attendant

5. CHW

4. Matron

3. Midwife

2. Nurse

1. Doctor

99. No Response

88. Do not Know

7. Other-specify (253S. Specify (String 100))

254. Where did the check take place?

- If primary health center, SPECIFY which one

6. Dispenp / maternity

5. Community referral hospital (Bankass)

4. Regional hospital

3. National hospital

2. Other home

1. Her home

10. Office of private care

9. Private Clinic / Hospital

8. Other public sector

7. Primary health center-specify (254S. Specify (String 100))

12. Pharmacy

15. Other-specify (254S. Specify (String 100))

14. Transit

13. Other private sector

11. Treatment Room

99. No Response

88. Do not Know

255. How long after delivery did the first check take place?

- - ENTER value
  - If unknown, ENTER “88”
  - If no response, ENTER “99”

0-99

- - ENTER unit

3. Weeks

2. Days

1. Hours

256. [NAME] = RESPONSE TO 231

In the two months after [NAME] was born, did a health care provider or a traditional birth attendant check on [NAME]’s health?

3. No

2. Yes, traditional midwife

1. Yes, health professional (doctor, nurse, midwife)

🡪GO TO 257 🡪GO TO 257 🡪GO TO 260

9. No Response

8. Do not Know

🡪GO TO 260 🡪GO TO 260

257. [NAME] = RESPONSE TO 231

How long after delivery was [NAME]’s health first checked?

- - ENTER value
  - If unknown, ENTER “88”
  - If no response, ENTER “99”

0-99

- - ENTER unit

Weeks

Days

Hours

258. Who checked on (NAME)’s health at that time?

- - RECORD ALL MENTIONED
  - PROBE for most qualified person

1. Doctor

6. Traditional birth attendant

5. CHW

4. Matron

3. Midwife

2. Nurse

7. Other-specify (253S. Specify (String 100))

99. No Response

88. Do not Know

259. Where did the check take place?

- - If primary health center, SPECIFY which one

1. Her home

5. District Hospital

4. Regional hospital

3. National hospital

2. Other home

8. Pharmacy

7. Private Clinic / Hospital

6. Primary health center-specify (259S. Specify (String 100))

99. No Response

88. Do not Know

9. Other-specify (259S. Specify (String 100))

260. [NAME] = RESPONSE TO 231

Did you ever breastfeed [NAME]?

9. No Response

8. Do not Know

1. Yes

2. No

🡪GO TO 301 🡪GO TO 301 🡪GO TO 301

↓

261. [NAME] = RESPONSE TO 231

Are you still breastfeeding [NAME]?

9. No Response

2. No

1. Yes

262. [NAME] = RESPONSE TO 231

If not, for how long did you breastfeed [NAME]?

- ENTER value

0-99

- ENTER unit

3. Year

2. Month

1. Day

263. For how long did you only breastfeed (NAME), that is to say, without other things to drink?

- ENTER value

0-99

- ENTER unit

3. Year

2. Month

1. Day

**Section 3: Children, Survival and Mortality**

301. Now I would like to ask about all the births you have had during your life. Have you ever given birth?

2. No

1. Yes

🡪GO TO 303

↓

302. How many of the children to whom you gave birth are still alive?

0-20

303. Have you ever given birth to a boy or girl who was born alive but later died?

- IF NO, PROBE: Any baby who cried, who made any movement, sound, or effort to breathe, or who showed any other signs of life even if for a very short time?

9. No Response

1. Yes

2. No

🡪GO TO 305 🡪GO TO 305

↓

304. How many children have died?

0-20

305. ADD ANSWERS TO 302 AND 304, AND ENTER THE TOTAL.

- IF NONE, RECORD '00'.

0-20

306A. [_____] = RESPONSE TO 305

Just to make sure that I have this right: you have had in TOTAL [_____] births during your life. Is that correct?

2. No

1. Yes

🡪GO TO 307

↓

306B. If Q306A = no, enter the correct number of total births

0-20

306C. If Q306A = no, enter the correct number of births of children still alive

0-20

306D. If Q306A = no, enter the correct number of children who died

0-20

307INTRO. Now I would like to record the names of all your births, whether still alive or not, starting with the first one you had.

- RECORD NAMES OF ALL THE BIRTHS IN 212.
- RECORD TWINS AND TRIPLETS AS SEPARATE.
- ENTER [1] to continue

1. Continue

Birth History Loop (Max = 30)

307. What name was given to your [first / next] baby?

- ENTER THE NAME.

NAME (String 100)

308. [NAME] = RESPONSE TO 307

On what day, month, and year was [NAME] born?

- ENTER day
- If unknown, ENTER “88”
- If no response, ENTER “99”

01-31, 88, 99

- ENTER month
- If unknown, ENTER “88”
- If no response, ENTER “99”

01-12, 88, 99

- ENTER year
- If unknown, ENTER “8888”
- If no response, ENTER “9999”

1900-2100, 8888, 9999

309. [NAME] = RESPONSE TO 307

Is [NAME] a boy or girl?

2. Girl

1. Boy

310. [NAME] = RESPONSE TO 307

Is [NAME] still alive?

2. No

1. Yes

🡪GO TO 312

↓

311. [NAME] = RESPONSE TO 307

IF ALIVE: How old was [NAME] at [NAME]’s last birthday?

- Enter the age in years, months and days.
- All fields are required.
- Mark 00 years if the child is less than one year, 00 months if the child is less than one month
- ENTER years:
- If unknown, ENTER “88”
- If no response, ENTER “99”

0-40, 88, 99

- ENTER months:
- If unknown, ENTER “88”
- If no response, ENTER “99”

0-12, 88, 99

- ENTER days:
- If unknown, ENTER “88”
- If no response, ENTER “99”

0-31, 88, 99

312. [NAME] = RESPONSE TO 307

IF DECEASED: What was the date of death of [NAME]?

- Mark MONTH and YEAR. The two fields are mandatory
- ENTER month as “MM”
- If unknown, ENTER “88”
- If no response, ENTER “99”

01-12, 88, 99

- ENTER year as “YYYY”
- If unknown, ENTER “8888”
- If no response, ENTER “9999”

1900-2100, 8888, 9999

313. [NAME] = RESPONSE TO 307, HE/SHE = [BOY / GIRL]

IF DECEASED: How old was [NAME] when [he / she] died?

- Enter the age in years, months and days.
- All fields are required.
- Mark 00 years if the child was less than one year, 00 months if the child was less than one month
- ENTER years:
- If unknown, ENTER “88”
- If no response, ENTER “99”

0-40, 88, 99

- ENTER months:
- If unknown, ENTER “88”
- If no response, ENTER “99”

0-12, 88, 99

- ENTER days:
- If unknown, ENTER “88”
- If no response, ENTER “99”

0-31, 88, 99

314. [NAME OF PREVIOUS BIRTH] = RESPONSE TO 307_X-1 [NAME] = RESPONSE TO 307_X

Were there any other live births between [NAME OF PREVIOUS BIRTH] and [NAME], including any children who died after birth?

2. No

1. Yes

End of Birth History Loop

315CKPT. WHETHER NUMBER OF BIRTHS IS CORRECT

|  |  |  |  |
| --- | --- | --- | --- |
|  | 1. RECORDED NUMBER OF BIRTHS IS INCONSISTENT WITH LOOPED RESPONSES | 1. RECORDED NUMBER OF BIRTHS IN TABLE IS CONSISTENT WITH RESPONSES IN LOOP | → GO TO 301 |
|  | ↓ |  |  |

COMPARE WITH THE NUMBER OF BIRTHS RECORDED IN THE TABLE ABOVE AND MARK:

NUMBERS ARE EQUAL? DIFFERENT (PROBE AND CORRECT)?

**Section 4: Diseases Of Children**

**Diarrhea**

401. [NAME]

Has [NAME] had diarrhea in the last 2 weeks?

9. No Response

1. Yes

2. No

8. Do not Know

🡪GO TO 500 🡪GO TO 500 🡪GO TO 500

402. Was there blood in the stool?

9. No Response

8. Do not Know

2. No

1. Yes

403. Did you seek advice or treatment for the diarrhea from any source?

9. No Response

8. Do not Know

2. No

1. Yes

🡪GO TO 405

↓ ↓ ↓

404. Why did you not get advice or treatment?

- PROBE: Other reasons?
- RECORD ALL MENTIONED

🡪GO TO 500

2. I could not cross the distance to the health center

3. I do not make decisions about seeking health care

1. I could not pay the fee

4. I arrived at the health center, but there was no equipment, material, or infrastructure to give me care

6. I arrived at the health center, but I did not find staff qualified enough to give me care

5. The prescribed medication was not available

9. I did not have enough time

8. I did not think it was serious enough

7. I did not know what to do

99. No Response

88. Do not Know

10. Other-specify (404S. Specify (String 100))

405. Where did you seek advice or treatment? Please list all sources in the order that you got them.

- PROBE: Anywhere else?
- PROBE TO IDENTIFY THE SOURCE
- If primary health center, SPECIFY which one

4. Community referral hospital (Bankass)

3. Regional hospital

2. National hospital

1. At my house

9. Home of CHW

8. At CHW

7. Pharmacy

6. Private Clinic / Hospital

5. Primary health center-specify (405S. Specify (String 100))

13. Other-specify (405S. Specify (String 100))

12. Shop

11. At home, a travelling seller

10. At a traditional healer

99. No Response

88. Do not Know

406. HE/SHE = [BOY / GIRL]

Did someone evaluate the child before [he / she] went to the health center? If so, who was it?

3. Other women

2. Husband

1. Myself

0. No

🡪GO TO 410 🡪GO TO 410 🡪GO TO 410 🡪GO TO 410

4. Other close relatives

7. Doctor

6. CHW

5. Friends / neighbors

🡪GO TO 410 🡪GO TO 410 🡪GO TO 407 🡪GO TO 410

11. Traditional healer

10. Matron

9. Midwife

8. Nurse

🡪GO TO 410 🡪GO TO 410 🡪GO TO 410 🡪GO TO 410

99. No Response

88. Do not Know

12. Drug Seller

🡪GO TO 410 🡪GO TO 410 🡪GO TO 410

407. Where was the evaluation conducted?

3. Other-specify (407S. Specify (String 100))

2. CHW Site

1. At home

🡪GO TO 408 🡪GO TO 409 🡪GO TO 409

8. Do not Know

9. No Response

🡪GO TO 409 🡪GO TO 409

408. How much time does it seem to take the CHW to arrive at your house?

99. No Response

88. Do not Know

5. Very short

4. Short

3. Normal

2. Long

1. Very long

409. How long did the evaluation last?

3. 2 hours (time between salifana and star)

2. 1 hour (time between fitri and saafo)

1. Less than an hour (less than the time between fitri and saafo)

99. No Response

88. Do not Know

6. 2 or more days

5. 1 day

4. ½ day

409CKPT.

🡪GO TO 414

↓

2. If 405=1, 8, 9, 88 OR 99

1. If 405=2-7 OR 10-13

410. How much time did it seem to take to get to the health center?

- If you went to several health centers, this is the last facility you attended.

88. Do not Know

5. Very short

99. No Response

4. Short

3. Normal

2. Long

1. Very long

411. At the health center, how long did it take before you received treatment?

2. 1 hour (time between fitri and saafo)

1. Less than an hour (less than the time between fitri and saafo)

99. No Response

88. Do not Know

6. 2 or more days

5. 1 day

4. ½ day

3. 2 hours (time between salifana and star)

412. How did the waiting time seem to you?

99. No Response

88. Do not Know

5. Very short

4. Short

3. Normal

2. Long

1. Very long

413. How long did the medical consultation take?

2. 1 hour (time between fitri and saafo)

1. Less than an hour (less than the time between fitri and saafo)

99. No Response

88. Do not Know

6. 2 or more days

5. 1 day

4. ½ day

3. 2 hours (time between salifana and star)

414. Did you take time off work to bring your child to the clinic? That is to say, the work you do that generates income. If so, how many days?

- If no leave taken, ENTER 0
- If unknown, ENTER 88
- If no response, ENTER 99

0-99

415. While seeking care for your child, which of the following expenses did you obtain?

1. Medication / treatment

9. No Response

8. Do not Know

2. No

1. Yes

2. Laboratory / diagnostic tests

9. No Response

8. Do not Know

2. No

1. Yes

3. Other health expenses

9. No Response

8. Do not Know

2. No

1. Yes

4. Food / Accommodation

9. No Response

8. Do not Know

2. No

1. Yes

5. Other expenses

9. No Response

8. Do not Know

2. No

1. Yes

415CKPT.

🡪GO TO 417

↓

2. If ANY 415==2, 8, or 9

1. If ALL 415==1

416. If Yes: How much did you pay for all these things?

- ENTER value in FCFA
- If unknown, ENTER 8888
- If no response, ENTER 9999

0-9999

417. What treatment did your child receive?

- RECORD ALL MENTIONED

4. Zinc tablet or syrup

3. Antimotility tablet or syrup

2. Antibiotic tablet or syrup

1. ORS

8. Unknown injection

7. Non-injectable antibiotic

6. Injectable antibiotic

5. Other / unknown tablet or syrup

11. Other-specify (417S. Specify (String 100))

10. Home remedy / herbal medicine

9. (IV) Intravenous

99. No Response

88. Do not Know

418a A

418a_1. [NAME]

How long after the onset of diarrhea, did [NAME] start taking the ORS?

88. Do not Know

3. Three days or more after diarrhea

2. Two days after diarrhea

1. Next day

0. Same day

99. No Response

418a_2. [NAME]

How long after the onset of diarrhea, did [NAME] start taking the antibiotic tablet or syrup?

88. Do not Know

3. Three days or more after diarrhea

2. Two days after diarrhea

1. Next day

0. Same day

99. No Response

418a_3. [NAME]

How long after the onset of diarrhea, did [NAME] start taking the antimotility tablet or syrup?

88. Do not Know

3. Three days or more after diarrhea

2. Two days after diarrhea

1. Next day

0. Same day

99. No Response

418a_4. [NAME]

How long after the onset of diarrhea, did [NAME] start taking the zinc tablet or syrup?

88. Do not Know

3. Three days or more after diarrhea

2. Two days after diarrhea

1. Next day

0. Same day

99. No Response

418a_5. [NAME]

How long after the onset of diarrhea, did [NAME] start taking the other / unknown tablet or syrup?

88. Do not Know

3. Three days or more after diarrhea

2. Two days after diarrhea

1. Next day

0. Same day

99. No Response

418a_6. [NAME]

How long after the onset of diarrhea, did [NAME] start taking the injectable antibiotic?

88. Do not Know

3. Three days or more after diarrhea

2. Two days after diarrhea

1. Next day

0. Same day

99. No Response

418a_7. [NAME]

How long after the onset of diarrhea, did [NAME] start taking the non-injectable antibiotic?

88. Do not Know

3. Three days or more after diarrhea

2. Two days after diarrhea

1. Next day

0. Same day

99. No Response

418a_8. [NAME]

How long after the onset of diarrhea, did [NAME] start taking the unknown injection?

88. Do not Know

3. Three days or more after diarrhea

2. Two days after diarrhea

1. Next day

0. Same day

99. No Response

418a_9. [NAME]

How long after the onset of diarrhea, did [NAME] start taking the (IV) intraveneous drug?

88. Do not Know

3. Three days or more after diarrhea

2. Two days after diarrhea

1. Next day

0. Same day

99. No Response

418a_10. [NAME]

How long after the onset of diarrhea, did [NAME] start taking the home remedy / herbal medicine?

88. Do not Know

3. Three days or more after diarrhea

2. Two days after diarrhea

1. Next day

0. Same day

99. No Response

418a_11. [NAME]

How long after the onset of diarrhea, did [NAME] start taking the other drug?

88. Do not Know

3. Three days or more after diarrhea

2. Two days after diarrhea

1. Next day

0. Same day

99. No Response

**Fever**

500. Has [NAME] been ill with a fever at any time during the last 2 weeks?

9. No Response

8. Do not Know

1. Yes

2. No

🡪GO TO 600 🡪GO TO 600 🡪GO TO 600

501. Did you seek advice or treatment for the fever from any source?

9. No Response

8. Do not Know

2. No

1. Yes

🡪GO TO 503 🡪GO TO 503 🡪GO TO 503

502. Why did you not get advice or treatment?

- PROBE: Other reasons?
- RECORD ALL MENTIONED

🡪GO TO 600

2. I could not cross the distance to the health center

3. I do not make decisions about seeking health care

1. I could not pay the fee

4. I arrived at the health center, but there was no equipment, material, or infrastructure to give me care

6. I arrived at the health center, but I did not find staff qualified enough to give me care

5. The prescribed medication was not available

9. I did not have enough time

8. I did not think it was serious enough

7. I did not know what to do

99. No Response

88. Do not Know

10. Other-specify (502S. Specify (String 100))

503. Where did you seek advice or treatment? Please list all sources in the order that you got them.

- PROBE: Anywhere else?
- PROBE TO IDENTIFY THE SOURCE
- If primary health center, SPECIFY which one

4. Community referral hospital (Bankass)

3. Regional hospital

2. National hospital

1. At my house

9. Home of CHW

8. At CHW

7. Pharmacy

6. Private Clinic / Hospital

5. Primary health center-specify (503S. Specify (String 100))

13. Other-specify (503S. Specify (String 100))

12. Shop

11. At home, a travelling seller

10. At a traditional healer

99. No Response

88. Do not Know

504. At any time during the illness, did (NAME) have blood taken from (NAME)’s finger or heel for testing? If so, who took the blood?

5. Friends / neighbors

4. Other close relatives

3. Other women

2. Husband

1. Myself

0. No

12. Drug Seller

10. Matron

11. Traditional healer

8. Nurse

9. Midwife

7. Doctor

6. CHW

99. No Response

88. Do not Know

505. Did someone evaluate the child before (he/she) went to the health center? If so, who was it?

3. Other women

2. Husband

1. Myself

0. No

🡪GO TO 509 🡪GO TO 509 🡪GO TO 509 🡪GO TO 509

7. Doctor

6. CHW

5. Friends / neighbors

4. Other close relatives

🡪GO TO 509 🡪GO TO 509 🡪GO TO 506 🡪GO TO 509

11. Traditional healer

10. Matron

9. Midwife

8. Nurse

🡪GO TO 509 🡪GO TO 509 🡪GO TO 509 🡪GO TO 509

99. No Response

88. Do not Know

12. Drug Seller

🡪GO TO 509 🡪GO TO 509 🡪GO TO 509

506. Where was the evaluation conducted?

3. Other-specify (506S. Specify (String 100))

2. CHW Site

1. At home

🡪GO TO 507 🡪GO TO 508 🡪GO TO 508

8. Do not Know

9. No Response

🡪GO TO 508 🡪GO TO 508

507. How much time does it seem to take the CHW to arrive at your house?

99. No Response

88. Do not Know

5. Very short

4. Short

3. Normal

2. Long

1. Very long

508. How long did the evaluation last?

2. 1 hour (time between fitri and saafo)

1. Less than an hour (less than the time between fitri and saafo)

99. No Response

88. Do not Know

6. 2 or more days

5. 1 day

4. ½ day

3. 2 hours (time between salifana and star)

509CKPT.

2. If 503=1, 8, 9, 88 OR 99

1. If 503=2-7 OR 10-13

🡪GO TO 513

↓

509. How much time did it seem to take to get to the health center?

- - If you went to several health centers, this is the last facility you attended.

99. No Response

88. Do not Know

5. Very short

4. Short

3. Normal

2. Long

1. Very long

510. At the health center, how long did it take before you received treatment?

2. 1 hour (time between fitri and saafo)

1. Less than an hour (less than the time between fitri and saafo)

99. No Response

88. Do not Know

6. 2 or more days

5. 1 day

4. ½ day

3. 2 hours (time between salifana and star)

511. How did the waiting time seem to you?

99. No Response

88. Do not Know

4. Short

5. Very short

3. Normal

2. Long

1. Very long

512. How long did the medical consultation take?

2. 1 hour (time between fitri and saafo)

1. Less than an hour (less than the time between fitri and saafo)

99. No Response

88. Do not Know

6. 2 or more days

5. 1 day

4. ½ day

3. 2 hours (time between salifana and star)

513. Did you take time off work to bring your child to the clinic? That is to say, the work you do that generates income. If so, how many days?

- If no leave taken, ENTER 0
- If unknown, ENTER 88
- If no response, ENTER 99

0-99

514. While seeking care for your child, which of the following expenses did you obtain?

1. Medication / treatment

9. No Response

8. Do not Know

2. No

1. Yes

2. Laboratory / diagnostic tests

9. No Response

8. Do not Know

2. No

1. Yes

3. Other health expenses

9. No Response

8. Do not Know

2. No

1. Yes

4. Food / Accommodation

9. No Response

2. No

8. Do not Know

1. Yes

5. Other expenses

9. No Response

8. Do not Know

2. No

1. Yes

514CKPT.

🡪GO TO 516

↓

2. If ANY 514==2, 8, or 9

1. If ALL 514==1

515. If Yes: How much did you pay for all these things?

- ENTER value in FCFA
- If unknown, ENTER 8888
- If no response, ENTER 9999

0-9999

516. What treatment did your child receive?

- RECORD ALL MENTIONED

6. Quinine injection

5. Quinine tablet

4. Artemisinin Combination Therapy (ACT)

3. Amodiaquine

2. Chloroquine

1. SP / Fansidar

11. Ibuprofen

10. Paracetamol

9. Other antipaludienne-specify (516S. Specify (String 100))

8. Artemether injection

7. Artesunate injection

99. No Response

88. Do not Know

11. Other-specify (516S. Specify (String 100))

Fever Medication Loop

517a. [NAME], [INSERT DRUG 516] = RESPONSE TO 516

How long after the onset of fever, did (NAME) start taking the [INSERT DRUG 516]?

- Ask this question for all medications selected in Q516

88. Do not Know

3. Three days or more after fever

2. Two days after fever

1. Next day

0. Same day

99. No Response

517aCKPT.

|  |  |  |  |
| --- | --- | --- | --- |
|  | 1. No more medications selected in 516 | 1. Another medication selected in 516 | → GO TO 517a |
|  | ↓ |  |  |

End of Loop

**Cough / Difficulty Breathing**

600. [NAME]

Has [NAME] been ill with a cough at any time during the last 2 weeks?

9. No Response

8. Do not Know

2. No

1. Yes

601. [NAME]

When [NAME] was ill with a cough, did [NAME] have fast, short, rapid breaths or difficulty breathing at any time?

9. No Response

8. Do not Know

2. No

1. Yes

602. Did you seek advice or treatment for the diarrhea from any source?

9. No Response

8. Do not Know

2. No

1. Yes

🡪GO TO 604

↓ ↓ ↓

603. Why did you not get advice or treatment?

- PROBE: Other reasons?
- RECORD ALL MENTIONED

🡪GO TO 700a

2. I could not cross the distance to the health center

3. I do not make decisions about seeking health care

1. I could not pay the fee

4. I arrived at the health center, but there was no equipment, material, or infrastructure to give me care

6. I arrived at the health center, but I did not find staff qualified enough to give me care

5. The prescribed medication was not available

9. I did not have enough time

8. I did not think it was serious enough

7. I did not know what to do

99. No Response

88. Do not Know

10. Other-specify (603S. Specify (String 100))

604. Where did you seek advice or treatment? Please list all sources in the order that you got them.

- PROBE: Anywhere else?
- PROBE TO IDENTIFY THE SOURCE
- If primary health center, SPECIFY which one

4. Community referral hospital (Bankass)

3. Regional hospital

2. National hospital

1. At my house

9. Home of CHW

8. At CHW

7. Pharmacy

6. Private Clinic / Hospital

5. Primary health center-specify (604S. Specify (String 100))

88. Do not Know

13. Other-specify (604S. Specify (String 100))

12. Shop

11. At home, a travelling seller

10. At a traditional healer

99. No Response

605. HE/SHE = [BOY / GIRL]

Did someone evaluate the child before [he / she] went to the health center? If so, who was it?

3. Other women

2. Husband

1. Myself

0. No

🡪GO TO 609 🡪GO TO 609 🡪GO TO 609 🡪GO TO 609

7. Doctor

6. CHW

5. Friends / neighbors

4. Other close relatives

🡪GO TO 609 🡪GO TO 609 🡪GO TO 606 🡪GO TO 609

11. Traditional healer

10. Matron

9. Midwife

8. Nurse

🡪GO TO 609 🡪GO TO 609 🡪GO TO 609 🡪GO TO 609

99. No Response

88. Do not Know

12. Drug Seller

🡪GO TO 609 🡪GO TO 609 🡪GO TO 609

606. Where was the evaluation conducted?

9. No Response

8. Do not Know

3. Other-specify (606S. Specify (String 100))

2. CHW Site

1. At home

🡪GO TO 608

↓ ↓ ↓ ↓

607. How much time does it seem to take the CHW to arrive at your house?

99. No Response

88. Do not Know

5. Very short

4. Short

3. Normal

2. Long

1. Very long

608. How long did the evaluation last?

2. 1 hour (time between fitri and saafo)

1. Less than an hour (less than the time between fitri and saafo)

99. No Response

88. Do not Know

6. 2 or more days

5. 1 day

4. ½ day

3. 2 hours (time between salifana and star)

608CKPT.

🡪GO TO 613

↓

2. If 604=1, 8, 9, 88 OR 99

1. If 604=2-7 OR 10-13

609. How much time did it seem to take to get to the health center?

- - If you went to several health centers, this is the last facility you attended.

99. No Response

88. Do not Know

5. Very short

4. Short

3. Normal

2. Long

1. Very long

610. At the health center, how long did it take before you received treatment?

2. 1 hour (time between fitri and saafo)

1. Less than an hour (less than the time between fitri and saafo)

99. No Response

88. Do not Know

6. 2 or more days

5. 1 day

4. ½ day

3. 2 hours (time between salifana and star)

611. How did the waiting time seem to you?

99. No Response

88. Do not Know

5. Very short

4. Short

3. Normal

2. Long

1. Very long

612. How long did the medical consultation take?

2. 1 hour (time between fitri and saafo)

1. Less than an hour (less than the time between fitri and saafo)

99. No Response

88. Do not Know

6. 2 or more days

5. 1 day

4. ½ day

3. 2 hours (time between salifana and star)

613. Did you take time off work to bring your child to the clinic? That is to say, the work you do that generates income. If so, how many days?

- If no leave taken, ENTER 0
- If unknown, ENTER 88
- If no response, ENTER 99

0-99

614. While seeking care for your child, which of the following expenses did you obtain?

1. Medication / treatment

9. No Response

8. Do not Know

2. No

1. Yes

2. Laboratory / diagnostic tests

9. No Response

8. Do not Know

2. No

1. Yes

3. Other health expenses

9. No Response

8. Do not Know

2. No

1. Yes

4. Food / Accommodation

9. No Response

2. No

8. Do not Know

1. Yes

5. Other expenses

9. No Response

8. Do not Know

2. No

1. Yes

614CKPT.

🡪GO TO 616

↓

2. If ANY 614==2, 8, or 9

1. If ALL 614==1

615. If Yes: How much did you pay for all these things?

- ENTER value in FCFA
- If unknown, ENTER 8888
- If no response, ENTER 9999

0-9999

616. What treatment did your child receive?

- RECORD ALL MENTIONED

2. Antitussive

2. Another antibiotic-specify (616S. Specify (String 100))

1. Amoxicillin

5. Antimalarial

4. Local applications (ie, cream, ointment, butter)

3. Decongestant (taste in the nostrils)

99. No Response

88. Do not Know

8. Other-specify (616S. Specify (String 100))

7. Ibuprofen

6. Paracetamol

617a. [NAME], [INSERT DRUG 516] = RESPONSE TO 616

How long after the onset of fever, did (NAME) start taking the [INSERT DRUG 616]?

99. No Response

88. Do not Know

3. Three days or more after fever

2. Two days after fever

1. Next day

0. Same day

**Other diseases**

700a. [NAME]

Has [NAME] had any other types of illnesses or injuries the last two weeks?

9. No Response

8. Do not Know

2. No

1. Yes

700b. If so, what type of illness or injury?

4. Conjunctivitis (eye infection)

3. Bilharziose (blood in urine)

2. Intestinal parasite

1. Skin infection (skin)

7. Other-specify (700bS. Specify (String 100))

6. Wounds

5. Fractures (bone)

701. Did you seek advice or treatment for the diarrhea from any source?

9. No Response

8. Do not Know

2. No

1. Yes

🡪GO TO 703

↓ ↓ ↓

702. Why did you not get advice or treatment?

- PROBE: Other reasons?
- RECORD ALL MENTIONED

🡪GO TO 800

2. I could not cross the distance to the health center

3. I do not make decisions about seeking health care

1. I could not pay the fee

4. I arrived at the health center, but there was no equipment, material, or infrastructure to give me care

6. I arrived at the health center, but I did not find staff qualified enough to give me care

5. The prescribed medication was not available

9. I did not have enough time

8. I did not think it was serious enough

7. I did not know what to do

99. No Response

88. Do not Know

10. Other-specify (702S. Specify (String 100))

703. Where did you seek advice or treatment? Please list all sources in the order that you got them.

- PROBE: Anywhere else?
- PROBE TO IDENTIFY THE SOURCE
- If primary health center, SPECIFY which one

4. Community referral hospital (Bankass)

3. Regional hospital

2. National hospital

1. At my house

9. Home of CHW

8. At CHW

7. Pharmacy

6. Private Clinic / Hospital

5. Primary health center-specify (703S. Specify (String 100))

88. Do not Know

13. Other-specify (703S. Specify (String 100))

12. Shop

11. At home, a travelling seller

10. At a traditional healer

99. No Response

704. HE/SHE = [BOY / GIRL]

Did someone evaluate the child before [he/she] went to the health center? If so, who was it?

3. Other women

2. Husband

1. Myself

0. No

🡪GO TO 708 🡪GO TO 708 🡪GO TO 708 🡪GO TO 708

7. Doctor

6. CHW

5. Friends / neighbors

4. Other close relatives

🡪GO TO 708 🡪GO TO 708 🡪GO TO 705 🡪GO TO 708

11. Traditional healer

10. Matron

9. Midwife

8. Nurse

🡪GO TO 708 🡪GO TO 708 🡪GO TO 708 🡪GO TO 708

99. No Response

88. Do not Know

12. Drug Seller

🡪GO TO 708 🡪GO TO 708 🡪GO TO 708

705. Where was the evaluation conducted?

9. No Response

8. Do not Know

3. Other-specify (705S. Specify (String 100))

2. CHW Site

1. At home

🡪GO TO 707

↓ ↓ ↓ ↓

706. How much time does it seem to take the CHW to arrive at your house?

2. Long

99. No Response

88. Do not Know

5. Very short

4. Short

3. Normal

1. Very long

707. How long did the evaluation last?

2. 1 hour (time between fitri and saafo)

1. Less than an hour (less than the time between fitri and saafo)

99. No Response

88. Do not Know

5. 1 day

6. 2 or more days

4. ½ day

3. 2 hours (time between salifana and star)

707CKPT.

🡪GO TO 712

↓

2. If 703=1, 8, 9, 88 OR 99

1. If 703=2-7 OR 10-13

708. How much time did it seem to take to get to the health center?

- - If you went to several health centers, this is the last facility you attended.

99. No Response

88. Do not Know

5. Very short

4. Short

2. Long

3. Normal

1. Very long

709. At the health center, how long did it take before you received treatment?

1. Less than an hour (less than the time between fitri and saafo)

2. 1 hour (time between fitri and saafo)

99. No Response

88. Do not Know

6. 2 or more days

5. 1 day

4. ½ day

3. 2 hours (time between salifana and star)

710. How did the waiting time seem to you?

99. No Response

88. Do not Know

5. Very short

4. Short

3. Normal

2. Long

1. Very long

711. How long did the medical consultation take?

2. 1 hour (time between fitri and saafo)

1. Less than an hour (less than the time between fitri and saafo)

99. No Response

88. Do not Know

6. 2 or more days

5. 1 day

4. ½ day

3. 2 hours (time between salifana and star)

712. Did you take time off work to bring your child to the clinic? That is to say, the work you do that generates income. If so, how many days?

- If no leave taken, ENTER 0
- If unknown, ENTER 88
- If no response, ENTER 99

0-99

713. While seeking care for your child, which of the following expenses did you obtain?

1. Medication / treatment

9. No Response

8. Do not Know

2. No

1. Yes

2. Laboratory / diagnostic tests

9. No Response

8. Do not Know

2. No

1. Yes

3. Other health expenses

9. No Response

8. Do not Know

2. No

1. Yes

4. Food / Accommodation

9. No Response

2. No

8. Do not Know

1. Yes

5. Other expenses

9. No Response

8. Do not Know

2. No

1. Yes

713CKPT.

🡪GO TO 715

↓

2. If ANY 713==2, 8, or 9

1. If ALL 713==1

714. If Yes: How much did you pay for all these things?

- ENTER value in FCFA
- If unknown, ENTER 8888
- If no response, ENTER 9999

0-9999

715. What treatment did your child receive?

- RECORD ALL MENTIONED

4. Ibuprofen

5. Medicinal Plant

3. Paracetamol

2. Antimalarial

1. Antibiotic

99. No Response

88. Do not Know

6. Another medicine-specify (715S. Specify (String 100))

Illness or Injury Medication Loop

716a. [NAME], [INSERT DRUG 715] = RESPONSE TO 715

How long after the illness or injury started did [NAME] start taking the [INSERT DRUG 715]?

- Ask this question for all medications selected in Q715

88. Do not Know

3. Three days or more after diarrhea

2. Two days after diarrhea

1. Next day

0. Same day

99. No Response

716aCKPT.

|  |  |  |  |
| --- | --- | --- | --- |
|  | 1. No more medications selected in 715 | 1. Another medication selected in 715 | → GO TO 716a |
|  | ↓ |  |  |

End of Loop

**Access to essential care for the respondent**

800. Have you had an illness or injury in the last six months?

- Clarification: unrelated to pregnancy or childbirth

1. Yes

9. No Response

8. Do not Know

2. No

🡪END OF INTERVIEW 🡪END OF INTERVIEW 🡪END OF INTERVIEW

↓

801. Did you seek advice or treatment for the diarrhea from any source?

1. Yes

9. No Response

8. Do not Know

2. No

🡪GO TO 803

↓ ↓ ↓

802. Why did you not get advice or treatment?

- PROBE: Other reasons?
- RECORD ALL MENTIONED

🡪END OF INTERVIEW

2. I could not cross the distance to the health center

3. I do not make decisions about seeking health care

1. I could not pay the fee

4. I arrived at the health center, but there was no equipment, material, or infrastructure to give me care

6. I arrived at the health center, but I did not find staff qualified enough to give me care

5. The prescribed medication was not available

9. I did not have enough time

8. I did not think it was serious enough

7. I did not know what to do

99. No Response

88. Do not Know

10. Other-specify (802S. Specify (String 100))

803. Where did you seek advice or treatment? Please list all sources in the order that you got them.

- PROBE: Anywhere else?
- PROBE TO IDENTIFY THE SOURCE
- If primary health center, SPECIFY which one

3. Regional hospital

4. Community referral hospital (Bankass)

2. National hospital

1. At my house

9. Home of CHW

8. At CHW

7. Pharmacy

6. Private Clinic / Hospital

5. Primary health center-specify (805S. Specify (String 100))

13. Other-specify (803S. Specify (String 100))

12. Shop

11. At home, a travelling seller

10. At a traditional healer

88. Do not Know

99. No Response

804. Did someone evaluate you before you went to the health center? If so, who was it?

3. Other women

2. Husband

1. Myself

0. No

🡪GO TO 808 🡪GO TO 808 🡪GO TO 808 🡪GO TO 808

7. Doctor

6. CHW

5. Friends / neighbors

4. Other close relatives

🡪GO TO 808 🡪GO TO 808 🡪GO TO 805 🡪GO TO 808

11. Traditional healer

10. Matron

9. Midwife

8. Nurse

🡪GO TO 808 🡪GO TO 808 🡪GO TO 808 🡪GO TO 808

99. No Response

88. Do not Know

12. Drug seller

🡪GO TO 808 🡪GO TO 808 🡪GO TO 808

805. Where was the evaluation conducted?

9. No Response

8. Do not Know

3. Other-specify (805S. Specify (String 100))

2. CHW Site

1. At home

🡪GO TO 807

↓ ↓ ↓ ↓

806. How much time did it seem to take the CHW to arrive at your house?

5. Very short

4. Short

3. Normal

2. Long

1. Very long

9. No Response

8. Do not Know

807. How long did the evaluation last?

2. 1 hour (time between fitri and saafo)

1. Less than an hour (less than the time between fitri and saafo)

99. No Response

88. Do not Know

6. 2 or more days

5. 1 day

4. ½ day

3. 2 hours (time between salifana and star)

807CKPT.

🡪GO TO 812

↓

2. If 803=1, 8, 9, 88 OR 99

1. If 803=2-7 OR 10-13

808. How much time did it seem to take to get to the health center?

- - If you went to several health centers, this is the last facility you attended.

9. No Response

8. Do not Know

5. Very short

4. Short

3. Normal

2. Long

1. Very long

809. At the health center, how long did you wait for care?

2. 1 hour (time between fitri and saafo)

1. Less than an hour (less than the time between fitri and saafo)

99. No Response

88. Do not Know

6. 2 or more days

5. 1 day

4. ½ day

3. 2 hours (time between salifana and star)

810. How did the waiting time seem to you?

9. No Response

8. Do not Know

4. Short

5. Very short

3. Normal

2. Long

1. Very long

811. How long did the medical consultation take?

2. 1 hour (time between fitri and saafo)

1. Less than an hour (less than the time between fitri and saafo)

99. No Response

88. Do not Know

6. 2 or more days

5. 1 day

4. ½ day

3. 2 hours (time between salifana and star)

812. Did you take time off from work to go to the clinic? That is to say, the work you do that generates income. If so, how many days?

- If no leave taken, ENTER 0
- If unknown, ENTER 88
- If no response, ENTER 99

0-99

813. While seeking care for your child, which of the following expenses did you obtain?

1. Medication / treatment

9. No Response

8. Do not Know

2. No

1. Yes

2. Laboratory / diagnostic tests

9. No Response

8. Do not Know

2. No

1. Yes

3. Other health expenses

9. No Response

8. Do not Know

2. No

1. Yes

4. Food / Accommodation

9. No Response

2. No

8. Do not Know

1. Yes

5. Other expenses

9. No Response

8. Do not Know

2. No

1. Yes

813CKPT.

🡪END OF INTERVIEW

↓

2. If ANY 813==2, 8, or 9

1. If ALL 813==1

814. If Yes: How much did you pay for all these things?

- ENTER value in FCFA
- If unknown, ENTER 8888
- If no response, ENTER 9999

0-9999

END OF INTERVIEW

Before submitting the survey, return the roster and ensure that the husband of the woman surveyed was identified if it is a usual resident of the household.
