# Supplementary material for: Women’s empowerment, intrahousehold influences, and health system design on modern contraceptive use in rural Mali: a multilevel analysis of cross-sectional survey data
Source: Reprod Health. 2021 Mar 3;18:55. doi: 10.1186/s12978-020-01061-z (PMC7931535; doi:10.1186/s12978-020-01061-z)
Supplement: Supplementary file 2 — Additional File 2: Supplementary tables 1 and 2 [file 12978_2020_1061_MOESM2_ESM.docx]

| Supplementary Table 1: Household level sample characteristics for women with and without main outcome data | | | | | |
| --- | --- | --- | --- | --- | --- |
|  | | Women with data on contraceptive use  N=13,983 | | Women missing data on contraceptive use  N=49 | |
| **Household level characteristic** | | **Frequency** | **%** | **Frequency** | **%** |
| Household size | |  |  |  |  |
|  | Median/IQR | 6 | 4, 9 | 5 | 3, 7 |
|  | Missing | 28 | 0.2 | 0 | 0.0 |
| Distance to health center | |  |  |  |  |
|  | <2 km | 2698 | 19.3 | 10 | 20.4 |
|  | 2-4.99 km | 3476 | 24.9 | 13 | 26.5 |
|  | 5-6.99 km | 3263 | 23.3 | 10 | 20.4 |
|  | 7-9.99 km | 2579 | 18.4 | 5 | 10.2 |
|  | >=10 | 1487 | 10.6 | 7 | 14.3 |
|  | Missing | 480 | 3.4 | 4 | 8.2 |
| Household wealth quintile^a^ | |  |  |  |  |
|  | Poorest | 2379 | 17.0 | 3 | 6.1 |
|  | Poor | 2587 | 18.5 | 4 | 8.2 |
|  | Middle | 2700 | 19.3 | 9 | 18.4 |
|  | Rich | 2983 | 21.3 | 13 | 26.5 |
|  | Richest | 3158 | 22.6 | 20 | 40.8 |
|  | Missing | 176 | 1.3 | 0 | 0.0 |
| Water and sanitation | |  |  |  |  |
|  | Unimproved toilet facilities | 7104 | 50.8 | 23 | 46.9 |
|  | Improved toilet facilities | 6825 | 48.8 | 26 | 53.1 |
|  | Missing | 54 | 0.4 | 0 | 0.0 |
|  | Unimproved water source | 6268 | 44.8 | 16 | 32.7 |
|  | Improved water source | 7713 | 55.2 | 33 | 67.4 |
|  | Missing | 2 | 0.0 | 0 | 0.0 |
| Food insecurity in past 30 days | |  |  |  |  |
|  | Little to no hunger in the household | 12239 | 87.5 | 41 | 83.7 |
|  | Moderate hunger in the household | 977 | 7.0 | 3 | 6.1 |
|  | Severe hunger in the household | 750 | 5.4 | 5 | 10.2 |
|  | Missing | 17 | 0.1 | 0 | 0.0 |
| CHW services available | |  |  |  |  |
|  | None | 9617 | 68.8 | 32 | 65.3 |
|  | Satellite village^b^ | 1901 | 13.6 | 7 | 14.3 |
|  | Posted village | 2465 | 17.6 | 10 | 20.4 |
| Health catchment area | |  |  |  |  |
|  | Dimbal | 3140 | 22.5 | 5 | 10.2 |
|  | Lessagou | 2194 | 15.7 | 1 | 2.0 |
|  | Doundé | 1752 | 12.5 | 4 | 8.2 |
|  | Ende | 728 | 5.2 | 0 | 0.0 |
|  | Soubala | 2406 | 17.25 | 1 | 2.0 |
|  | Kanibozon | 1430 | 10.2 | 18 | 36.7 |
|  | Koulongon | 2333 | 16.7 | 20 | 40.8 |
| *Notes*   1. Household wealth quintile excludes water, sanitation, and hygiene measures, which are reported separately. 2. Satellite villages are within the CHW’s catchment area, five kilometers or less of the village where the CHW has a fixed site (posted village). | | | | | |

| Supplementary Table 2: Individual level sample characteristics for women with and without main outcome data | | | | | |
| --- | --- | --- | --- | --- | --- |
|  | | Women with data on contraceptive use  N=13,983 | | Women missing data on contraceptive use  N=49 | |
| **Individual level characteristic** | | **Frequency** | **%** | **Frequency** | **%** |
| Age | |  |  |  |  |
|  | 15-19 | 1819 | 13.0 | 20 | 40.8 |
|  | 20-24 | 2370 | 17.0 | 7 | 14.3 |
|  | 25-29 | 2740 | 19.6 | 6 | 12.2 |
|  | 30-34 | 2443 | 17.5 | 4 | 8.2 |
|  | 35-39 | 1993 | 14.3 | 5 | 10.2 |
|  | 40-44 | 1498 | 10.7 | 1 | 2.0 |
|  | 45-49 | 1120 | 8.0 | 6 | 12.2 |
| Number of living children | |  |  |  |  |
|  | Median/IQR | 3 | 1, 5 | 1 | 0, 4 |
|  | None | 2360 | 16.9 | 16 | 32.7 |
|  | 1-2 | 3840 | 27.5 | 18 | 36.7 |
|  | 3-4 | 3670 | 26.3 | 4 | 8.2 |
|  | 5-6 | 2683 | 19.2 | 5 | 10.2 |
|  | 7+ | 1410 | 10.1 | 6 | 12.2 |
|  | Missing | 20 | 0.1 | 0 | 0.0 |
| Ethnicity | |  |  |  |  |
|  | Dogon | 12609 | 90.2 | 37 | 75.5 |
|  | Peulh | 1027 | 7.3 | 9 | 18.4 |
|  | Other | 347 | 2.5 | 3 | 6.1 |
| Religion | |  |  |  |  |
|  | Muslim | 13684 | 97.9 | 48 | 98.0 |
|  | Catholic | 181 | 1.3 | 0 | 0.0 |
|  | Other | 118 | 0.8 | 1 | 2.0 |
| Marital status | |  |  |  |  |
|  | Never married | 1167 | 8.4 | 12 | 24.5 |
|  | Divorced/widowed | 162 | 1.2 | 0 | 0.0 |
|  | Polygynous marriage | 5387 | 38.5 | 22 | 44.9 |
|  | Monogamous marriage | 7257 | 51.9 | 15 | 30.6 |
|  | Married, arrangement unspecified | 10 | 0.1 | 0 | 0.0 |
| Education | |  |  |  |  |
|  | None | 12473 | 89.2 | 42 | 85.7 |
|  | Primary | 1311 | 9.4 | 7 | 14.3 |
|  | Secondary or higher | 181 | 1.3 | 0 | 0.0 |
|  | Missing | 18 | 0.1 | 0 | 0.0 |
| Participates in paid labor | |  |  |  |  |
|  | No | 12066 | 86.3 | 45 | 91.8 |
|  | Yes | 1914 | 13.7 | 3 | 6.1 |
|  | Missing | 3 | 0.0 | 1 | 2.0 |
| Mobility | |  |  |  |  |
|  | Been to no place | 4758 | 34.0 | 9 | 18.4 |
|  | Been to some/all places but none alone | 2494 | 17.8 | 4 | 8.2 |
|  | Been to some places alone | 4066 | 29.1 | 28 | 57.1 |
|  | Been to all places alone | 2629 | 18.8 | 5 | 10.2 |
|  | Missing | 36 | 0.3 | 3 | 6.1 |
| Tolerant attitudes for spousal violence | |  |  |  |  |
|  | Never tolerated | 3845 | 27.5 | 8 | 16.3 |
|  | Sometimes tolerated | 5960 | 42.6 | 31 | 63.3 |
|  | Always tolerated | 3682 | 26.3 | 5 | 10.2 |
|  | Missing | 496 | 3.6 | 5 | 10.2 |
| Decision-making | |  |  |  |  |
|  | Not involved in any domains | 10168 | 72.7 | 42 | 85.7 |
|  | Involved in some domains | 2460 | 17.6 | 5 | 10.2 |
|  | Involved/independent in all domains | 1303 | 9.3 | 1 | 2.0 |
|  | Missing | 52 | 0.4 | 1 | 2.0 |
